# Dams trigger long-term river-floodplain decoupling in dynamic Andean foreland rivers

**DOI:** 10.1126/sciadv.aec8647

**Published:** 2026-08-28

**Authors:** Muriel Z. M. Brückner, Andrew P. Nicholas, Rolf Aalto, Philip J. Ashworth, Jim Best, Thomas Dunne, Renato Paes de Almeida

**Affiliations:** ^1^ Department of Geography, University of Exeter, Exeter, EX4 4RJ, UK.; ^2^ Department of Civil and Environmental Engineering, Center for Computation and Technology, Louisiana State University, Baton Rouge, LA 70803, USA.; ^3^ Department of Earth and Space Sciences, University of Washington, Seattle, WA 98195, USA.; ^4^ Department of Earth Science and Environmental Change, University of Illinois Urbana-Champaign, Urbana, IL 61801, USA.; ^5^ Departments of Geography and GIS, and Mechanical Science and Engineering, and Ven Te Chow Hydrosystems Laboratory, University of Illinois Urbana-Champaign, Urbana, IL 61801, USA.; ^6^ Bren School of Environmental Science and Management, University of California Santa Barbara, Santa Barbara, CA 93106, USA.; ^7^ Department of Sedimentary and Environmental Geology, University of São Paulo, São Paulo, 05508-220, Brazil.

## Abstract

Ongoing and planned hydroelectric dam construction in the Andes will alter water and sediment delivery to downstream rivers, with likely substantial impacts on river and floodplain morphology, hydrology, and habitats. These disturbances will be greatest along laterally dynamic rivers with strong current channel-floodplain coupling, such as Andean foreland rivers. Using a hydro-morphodynamic river model, we show that these floodplains are rapidly transformed through widespread vertical and lateral erosion with persisting long-term effects: Erosion creates secondary floodplains entrenched several meters below pre-dam levels within a century. This degradation of the channel-floodplain complex changes floodplain inundation patterns and frequency and disconnects riparian water bodies from the active river. Such decoupling will be most severe in rivers characterized by high sediment loads and lateral migration rates before dam construction. Our simulations reveal that predictive approaches that neglect geomorphic changes, including lateral river mobility, may be of limited utility in assessing dam impacts on the construction and functioning of wetland habitats.

## INTRODUCTION

Although hydroelectric dams are central to renewable energy production in many economies (*1*, *2*), they cause river fragmentation, reduce flood amplitudes, distort seasonal flow regimes (*3*–*5*), and reduce sediment supplies below dams by up to 80 to 100% (*6*–*8*). These changes trigger downstream geomorphic responses such as channel incision (*8*), reduced channel migration (*9*, *10*), and diminished downstream nutrient delivery (*11*), with associated impacts on ecosystems (*5*, *12*, *13*).

**Fig. 1.**
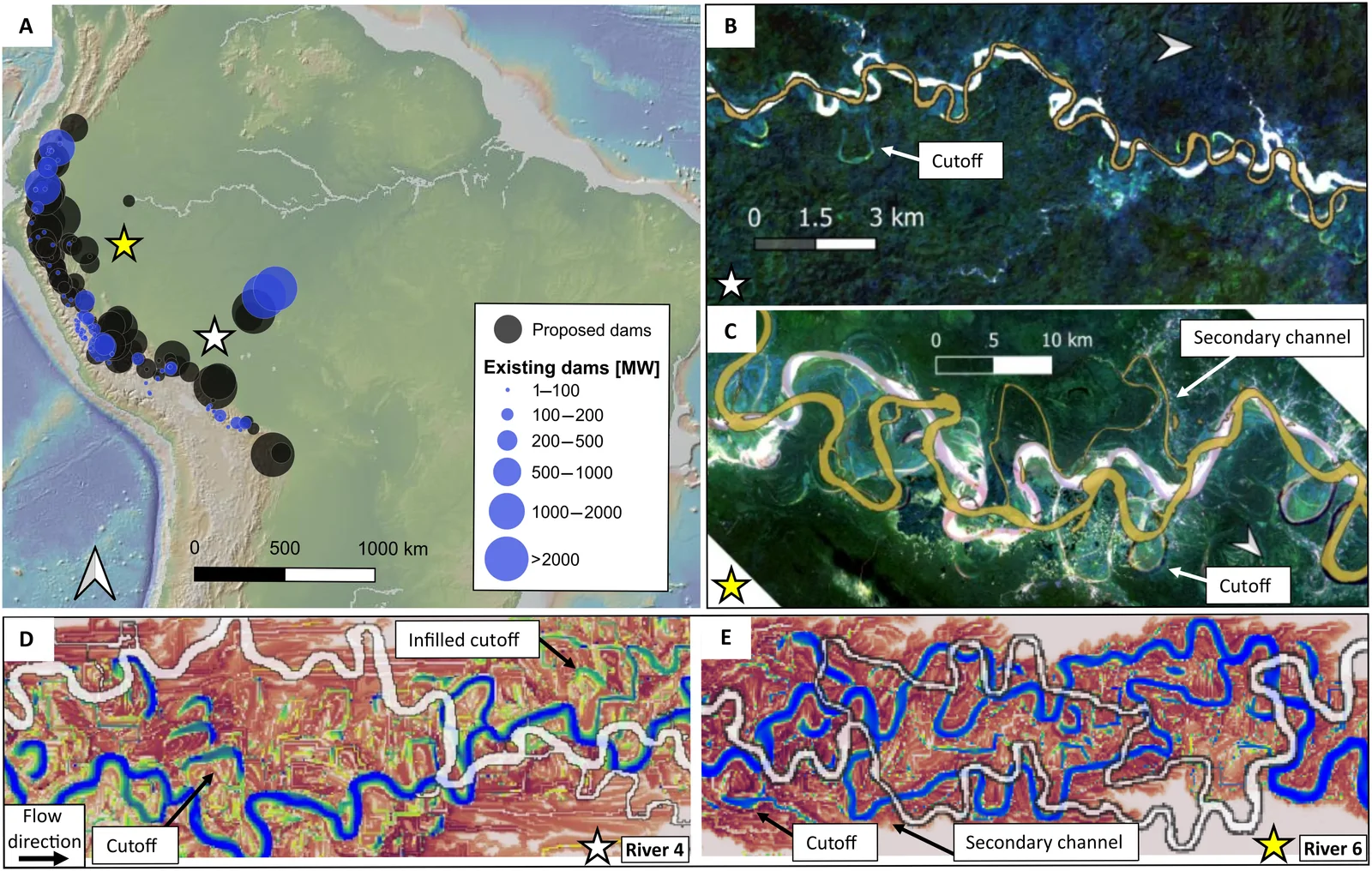
Locations of dams in the Amazon Basin and image of typical AFRs compared to topography for the two classes of modeled rivers. (**A**) Map (Source: Global Multi-Resolution DEM) of existing and proposed dam locations in the Andes and Madeira River (*4*). Circle sizes indicate dam capacity in megawatts (MW). (**B** and **C**) River channel outlines in 1987 (yellow) of the Beni and Ucayali rivers superimposed on Landsat imagery for 2023 [locations indicated by stars in (A)], with highlighted examples of abandoned channels (cutoffs). (**D** and **E**) Selected surface elevation maps, relative to the initial floodplain level, for modeled Rivers 4 and 6 shown at the end of the simulation (360 years) for the reference (no-dam) scenario (colored topography). White shaded channel indicates channel position 36 years earlier (star color indicates equivalent Landsat image for comparison), illustrating comparable migration behavior between model and observations [computed mean difference between modeled and observed, for Beni and Ucayali rivers, metrics: width-depth ratio = 17%; sinuosity = 12%; migration rate = 23% (*46*)]. Modeled river and floodplain morphologies exhibit meanders, multithread channels, and cutoff features common in AFR.

In rivers with laterally mobile channels, such as in the Amazon River Basin, channel-floodplain interactions control some of the world’s most ecologically sensitive floodplains (*2*, *14*). Although still at an early stage, planned dam construction in such environments will affect geomorphological processes that build floodplains and create habitat characteristics and functioning (*1*, *6*, *15*). In the Amazon Basin, 158 dams exist and 351 are planned (*4*, *16*), most notably in Andean headwater rivers (Fig. 1A), with impoundment water volumes surpassing tens of cubic kilometers (*9*). These Andean sand-bed rivers have single- or multithread meandering planforms characterized by intermediate channel gradients of ∼0.1 m/km and lateral migration rates averaging 30 m/year and ranging up to 150 m/year, with observed meander loop progression and frequent lake formation by channel cutoff (*17*, *18*). These channels are termed “white-water” rivers as a result of their high suspended sediment loads of several hundred megatons per year (*19*–*21*).

Despite the large-scale consequences of hydropower installations being highlighted qualitatively in a number of high-profile interdisciplinary reviews (*9*, *12*), no analytical approach has been proposed for quantitative assessments of floodplain habitat changes that are anticipated downstream of river impoundments. To address this problem, we have selected alluvial rivers draining from the Andes Range [Andean foreland rivers (AFR)] into the Amazon River, to guide a model-based analysis of the mechanisms by which dam construction causes substantial changes to river channels and floodplains and the complex aquatic and wetland habitats they support. AFR deliver 50% of the flow and 93% of the sediment with associated organic matter and nutrients received by the mainstem Amazon (*4*, *22*–*24*). They are laterally dynamic, resulting in important river-floodplain sediment exchanges (*25*) that create diverse floodplain morphologies (*17*, *20*). These include abandoned channel lakes that provide habitat to the world’s highest diversity of freshwater species (*12*, *26*). Impoundment will result not only in immediate impacts, such as declining sediment concentrations and lateral channel dynamics (*27*, *28*), and decreasing fishery yields (*29*, *30*) but also in additional long-term environmental changes (*31*) that now remain unquantified.

Previous analyses of the morphodynamic response of rivers to dams have largely concerned laterally confined, heavily engineered rivers and report channel incision or accretion, narrowing, sediment coarsening, reduced channel migration, and vegetation encroachment (*31*–*33*). Models investigating the impacts of dams in unconstrained dynamic rivers either simplify geomorphic change (*8*, *34*, *35*), ignore channel-floodplain coupling and lateral migration (*36*), or investigate annual to decadal timescales, thereby neglecting long-term geomorphic adaptations of the floodplains (*37*). Moreover, studies assessing the effects of dams on flood regimes and their role in maintaining floodplain habitats (*9*, *12*, *38*) disregard the role of the river in producing and maintaining critical floodplain water bodies, such as by channel migration and bend cutoff. These shortcomings limit current analyses of the mechanisms by which dam construction disrupts channel-floodplain interactions and habitat formation over large spatio-temporal scales that are beyond the scope of available monitoring records and instrumental studies.

To investigate these interactions and anticipate their long-term consequences, it is thus necessary to use models that reproduce the geomorphic behavior of river migration and floodplain construction. However, many existing models are limited in their capacity to represent key processes controlling coupled river-floodplain evolution, such as bank erosion, point-bar accretion, channel cutoffs, and avulsions (*39*, *40*). To simulate the response of dynamic AFR to dam construction over timescales of decades to centuries, we take advantage of a recently developed process-based hydro-morphodynamic model that reproduces realistic channel-floodplain dynamics, both qualitatively and quantitatively, in braided and meandering rivers, such as bend migration, vegetation establishment and mortality, and channel abandonment (*41*, *42*). The model is based on widely used physical and empirical relations for flow, suspended, and bedload sediment transport and includes primary processes controlling channel-floodplain coupling. We simulate six exploratory river reaches each ∼100 km in length. These exploratory models are widely used tools to assess first-order principles in complex morphodynamic systems [e.g., (*43*, *44*)]. Instead of tuning parameters to match short-term, site-specific observational datasets, exploratory models focus on fundamental process interactions (*45*), which allow an understanding of the broader feedback mechanisms that control long-term river behavior and drive geomorphic changes in response to dam construction. In the Amazon Basin, where long-term measurements of geomorphic change are absent, these types of models are necessary to quantify the foreseeable hydrogeomorphic responses of rivers to dam construction.

## RESULTS

First, we calibrated our models over a pre-dam period to represent two classes of dynamic AFR with a representative range of channel properties (width, depth, and sinuosity), migration rates, and geomorphic floodplain features observed along major AFRs. The river models were informed using data from the Beni River, Bolivia, and Ucayali River, Peru, for initial channel width and depth, grain size fractions, and water discharge at the upstream boundary. All rivers developed a realistic morphology from an initially straight 600-m-wide and 8-m-deep channel by simulating sediment transport, erosion, and deposition of four grainsize fractions between silt and gravel (table S1). Support for the accuracy of the model predictions comes from their conformity with observed channel behavior and floodplain features in Amazon Basin wetlands (Fig. 1 and fig. S1) (*17*, *20*, *46*–*48*).

We modeled six river reaches characterized by combinations of varying grain sizes, bank erodibility, initial channel slopes, and sediment transport rates. The reaches were classified on the basis of their planform and geomorphic behavior: Class 1 represents single-thread meandering rivers driven by higher channel gradients, sediment transport, and lateral migration rates (Rivers 1 to 4; e.g., Beni River). Class 2 represents multithread meandering planforms resulting from lower gradients, sediment transport, and lateral migration rates (Rivers 5 and 6; e.g., Ucayali River) [compare Fig. 1 (B and C with D and E); table S1]. These classes allow us to quantify the impacts of dams on dynamic rivers with varying lateral mobility and planform characteristics.

Following this pre-dam period, we modeled the hydro-morphodynamic adaptation of each river over 360 years for each river. This timescale provides evidence to quantify long-term landscape response to anthropogenic impacts by accounting for morphodynamic lag times and reequilibration of channel gradients and channel-floodplain interactions (*49*). We model two simulations per river, consisting of a reference scenario (without a dam) and a dam scenario, the latter with an adjusted flow hydrograph and sediment supply. We simulated several supply scenarios informed by past observations (see table S2) (*1*, *9*) and herein present results for a scenario with a 30% reduction in peak flows, 15% increase in base flow, and sediment supply reduced to 8 to 9% of the reference (no dam) scenario due to sediment trapping. The key trends in the results presented below are consistent for all scenarios modeled (see the Supplementary Materials).

### Impacts of dams on river morphology and dynamics

**Fig. 2.**
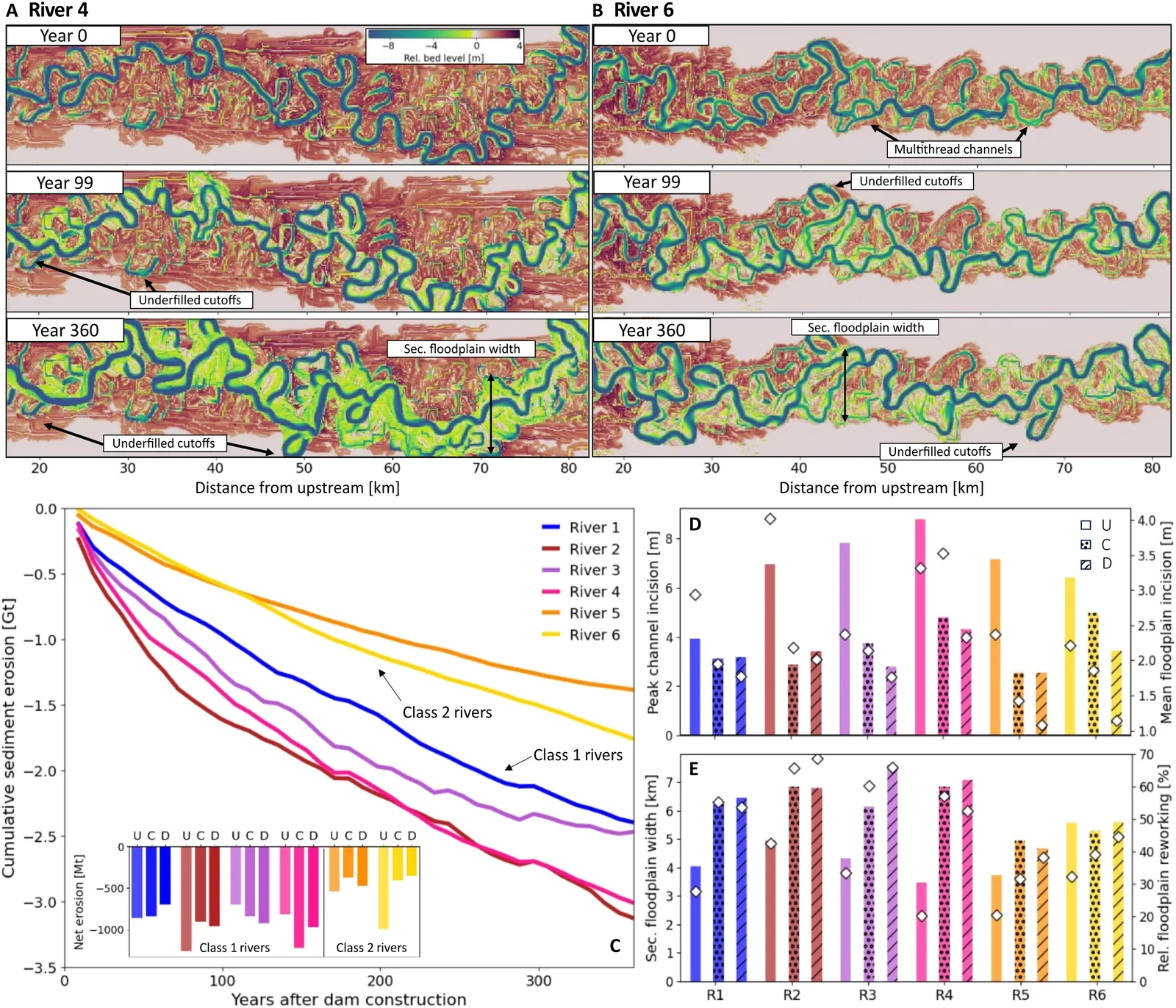
Geomorphic changes over the 360-year post-dam simulation period (dam scenario A) divided into 35-km-long upstream, central, and downstream reaches. (**A** and **B**) Relative surface elevation maps for Rivers 4 (class 1) and 6 (class 2) at three time steps after dam construction showing the formation of the secondary floodplain and disconnection of cutoff channels. (**C**) Cumulative erosion (lines) in each river with bar plots showing net erosion at the end of the simulation in equidistant upstream, central, and downstream reaches (U, C, and D). Lower cumulative erosion in class 2 rivers (Rivers 5 and 6) compared to the remaining simulated rivers is linked with their lower mobility. (**D**) Peak (95 percentile of the maximum) vertical incision of the channel bed (bars) and mean incision on the floodplain (diamond symbols) relative to the initial channel and floodplain levels. Values are averaged along rivers (R1 to R6) and reaches (U, C, and D). (**E**) Secondary floodplain width (bars) and mean floodplain reworking rate (diamond symbols) as a percentage of the reworking rate in the reference (no dam) scenario.

Our model results show how altered flow and sediment regimes below dams induce both vertical channel incision and lateral floodplain erosion in all rivers, creating a secondary floodplain that is 8 to 15 times the original mean channel width and several meters below the original floodplain level (Fig. 2, A to C, and table S1). Although rates of simulated channel incision after dam construction (2 to 5 m over 99 years close to the dam; Fig. 2D) are of the same order of magnitude as observations from other dammed rivers, such as the lower White River, USA (∼2 m over 42 years) (*50*, *51*), the lower American River, USA (∼3 m over 35 years) (*52*), the Arno River, Italy (2 to 9 m over 45 years) (*53*), or the Huang He River, China (>2 m over 20 years) (*54*), they are ∼50% lower. We attribute this to the absence of lateral mobility in many dammed rivers compared with our simulations: Lateral sediment supply from bank erosion balances incision rates and reduces vertical erosion in our simulations. Despite these conservative incision rates in our simulations, substantial erosion occurs rapidly—within 100 years of dam construction—before declining gradually (Fig. 2A and fig. S5). The net eroded volume depends on the degree of channel-floodplain coupling before damming: In rivers with low rates of sediment transport and migration (class 2, Rivers 5 and 6), net erosion is lower, and secondary floodplains are narrower than in more dynamic rivers due to lower initial slopes and lateral mobility (*55*). In contrast, in rivers with higher sediment transport and migration rates (class 1, Rivers 1 to 4), dams cause the creation of wide secondary floodplains and high net erosion.

Net peak erosion occurs in the upstream and central reaches of most rivers, while vertical channel incision is greatest near the dam (Fig. 2, C and D), with maximum channel bed lowering approaching 25 to 50% of pre-dam bankfull channel depths within the first century after impoundment and up to 65% by year 360. At greater distances from the dam, sediment transport fluxes are gradually enhanced by channel and floodplain erosion, channel incision is reduced, and natural channel-floodplain functions, such as overbank sedimentation, point bar deposition, lateral channel migration, and cutoff formation, are restored, albeit limited to the secondary floodplain. However, in all rivers, post-dam channel migration remains consistently below reference (i.e., no-dam) rates (Fig. 2E), illustrating the persistent long-term impacts of dams on fluvial morphodynamics. Although greater lateral floodplain reworking compensates for vertical incision downstream, it results in wider secondary floodplains with increasing distance from the dam.

### Hydrological consequences of dam-induced morphodynamic changes

**Fig. 3.**
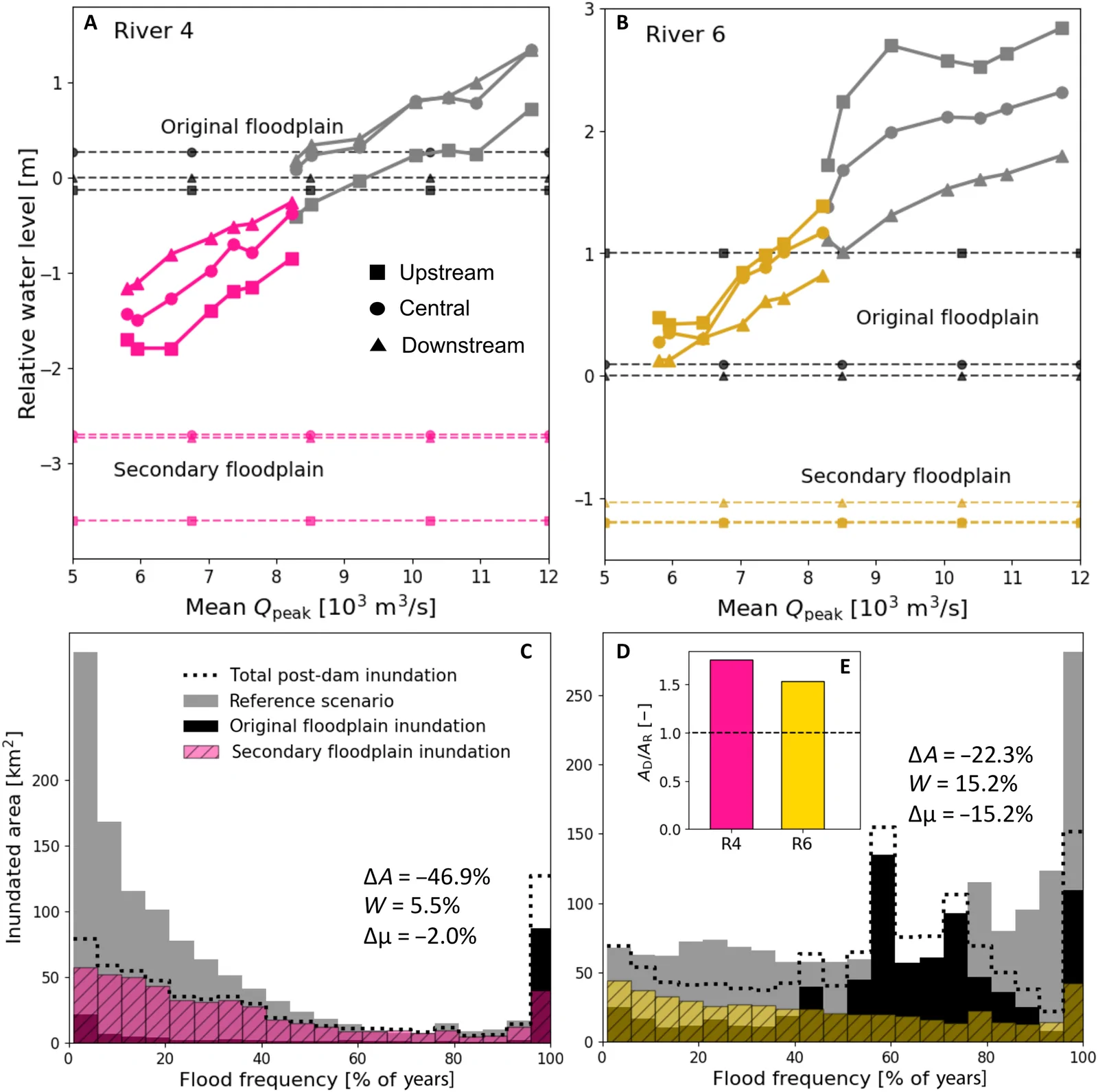
Hydrological changes following dam construction for Rivers 4 and 6. (**A** and **B**) Relationship between mean peak flood discharge and relative water level in the reference (no-dam, gray lines) and dam scenarios (colored lines) for a class 1 river (River 4) and class 2 river (River 6). Data are averaged over years 180 to 360 (solid lines) and each region (upstream, central and downstream) of the model domain (symbols). Vertical dashed lines represent the mean floodplain elevation of the original (non-reworked) floodplain (black lines) and secondary floodplain (colored lines) in each region. Water level is normalized by the mean secondary floodplain elevation in the downstream reach. (**C** and **D**) Area of floodplain inundation plotted as a function of flood frequency averaged over years 180 to 360 for Rivers 4 and 6 [colors as in (A) and (B)]. Gray bars indicate inundation in the no-dam scenario. Dotted line represents total floodplain inundation in dam scenarios, which is the sum of the inundation of the original (black bars) and secondary (colored bars) floodplains. Kolmogorov-Smirnoff tests showed significant differences (*P* < < 0.001) between total post-dam and reference inundation distributions. Δ*A* represents the difference in flooded area, *W* is the Wasserstein distance, and Δμ is the shift in the mean between total post-dam and reference inundation. (**E**) Ratio of unfilled cutoff area for Rivers 4 and 6 at the end of the dam scenario, *A*_D_, and the no-dam scenario, *A*_R_ [colors as in (A) and (B)].

Loss of floodplain area has been linked mainly with altered discharge regimes due to dam construction and operation (*38*, *56*). Here, we show that, following dam closure, channel-floodplain erosion combined with diminished high flow discharges transforms relationships between water level, discharge, and floodplain inundation. Stage-discharge relationships (Fig. 3, A and B) illustrate a progressive drop in mean water levels for similar peak discharges after dam closure, especially in reaches close to the dam where incision is largest. Projections from two modeled rivers exemplify how peak water levels for the dam scenarios fall by ∼0.5 to 1 m as compared to the reference (no-dam) cases for the same discharge, similar magnitudes as reported from field observations (*57*). Peak post-dam water levels continue to exceed the mean elevation of the new secondary floodplain but do not reach the higher original floodplain level in the mobile river 4, even for peak discharges greater than those modeled here (i.e., during exceptionally wet years). Lower water levels may also drive a disconnection of existing riparian water bodies (cutoff channels) from the active channel.

The combination of declining water levels and both incised channels and floodplains causes a statistically significant loss of floodplain inundation in all rivers (Fig. 3, C and D). Where initial sediment supply rates are high (class 1 rivers), inundation is restricted largely to the new secondary floodplain (Fig. 3C, colored shaded bars). In these rivers, the total inundated area is reduced by almost 50% after dam construction. Areas inundated at low to intermediate flood frequencies (<40% of years) are reduced significantly. In rivers with lower incision (class 2, e.g., River 6), this riparian disconnection is less pronounced with a total loss of 22% of the floodplain, but inundation occurs only in the central and downstream portions of the original floodplain or for the highest discharges (Fig. 3B). In these cases, the area inundated at both low and high flood frequencies is reduced, with a shift toward areas flooded at intermediate frequencies after dam construction (Fig. 3D). This disconnection promotes greater total cutoff area in dammed rivers, as compared to no-dam simulations (Fig. 3E), because cutoffs remain underfilled due to the reduced sediment delivery to the floodplain. These results illustrate the profound long-term impacts of dams on the connectivity between active river channels and their floodplains for the two classes of rivers.

### The role of initial river dynamics

**Fig. 4.**
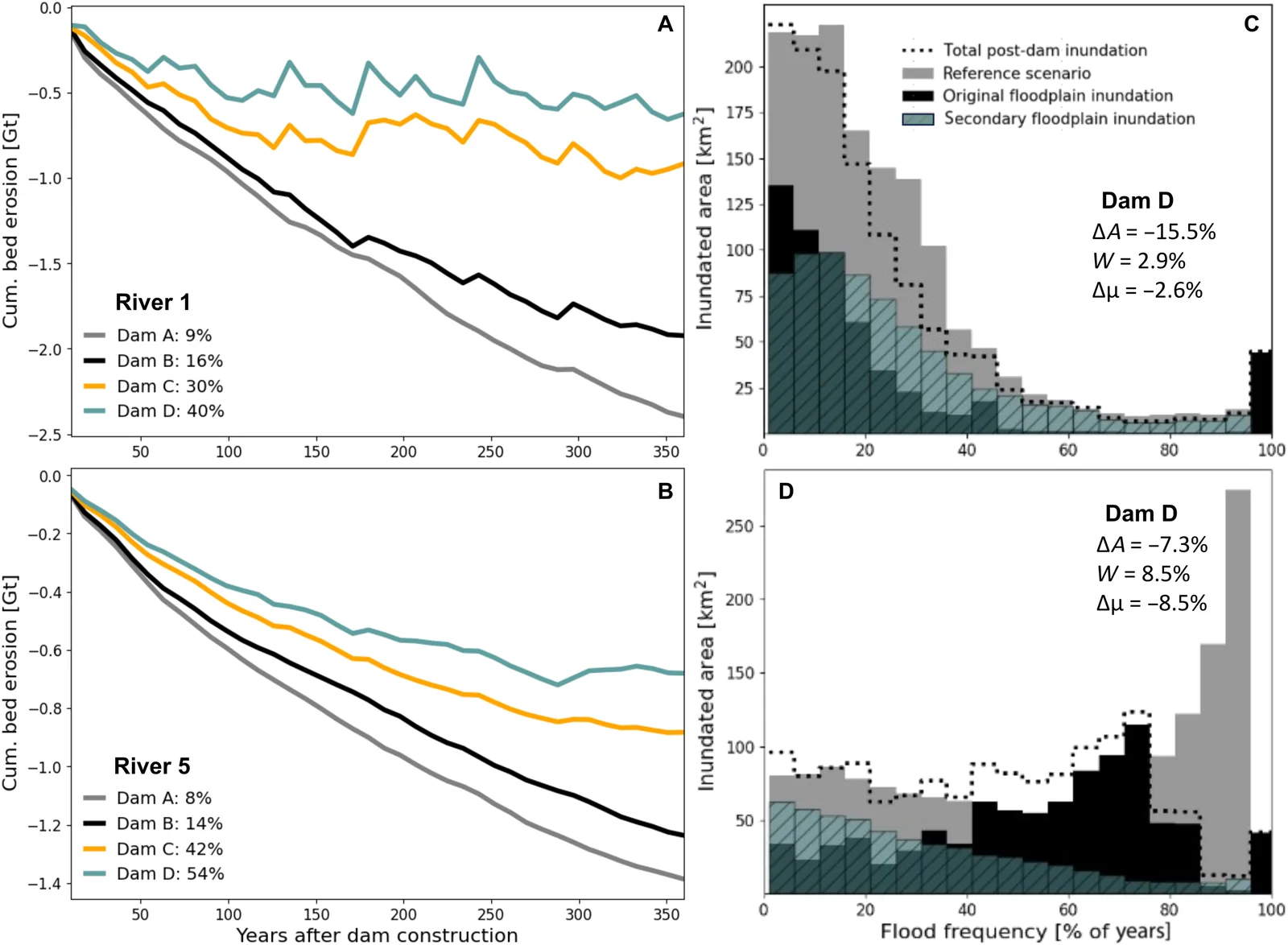
Cumulative erosion for four dam scenarios simulated for Rivers 1 and 5. (**A** and **B**) Cumulative erosion in Rivers 1 (class 1) and 5 (class 2) over time at 9-year intervals for increasing total sediment supply rates at the dam (A to D; percentages are total sediment supply compared to reference simulation). Although net erosion decreases with reduced trapping rates at the dam, substantial erosion occurs within the first 50 to 100 years in all scenarios, especially in River 5. Note that the percentages are variable between rivers as their calibrated sediment supply coefficients vary (Eq. 1). (**C** and **D**) Area of floodplain inundation plotted as a function of flood frequency averaged over years 180 to 360 for dam scenario D and the two rivers. Gray bars indicate inundation in the no-dam scenario. Dotted line represents total floodplain inundation in dam scenarios, which is the sum of the inundation of the original (black bars) and secondary (colored bars) floodplains. Kolmogorov-Smirnoff tests showed significant differences (*P* < < 0.001) between total post-dam and reference inundation distributions. Δ*A* represents the difference in total flooded area between post-dam and reference scenarios, *W* is Wasserstein distance and difference in mean flood frequency, and Δμ shows a shift between total post-dam and reference inundation toward reduced flood frequencies.

River response to damming depends strongly on pre-dam channel gradients, sediment transport rates, associated lateral migration rates, and degree of channel-floodplain coupling (Table 1). Thus, modeled rivers with higher lateral migration rates (Rivers 1 to 4) experience greater floodplain entrenchment and associated impacts on flood water levels, channel-floodplain connectivity, and floodplain inundation (Figs. 3 and 4). These characteristics also regulate the rate of floodplain lake production (by bend cutoff), as well as the exchanges of water, sediment, and potentially nutrients that control the physical and chemical characteristics, as well as residence time of water within these critical floodplain water bodies. Class 2 rivers with lower initial sediment transport rates (Rivers 5 and 6) show less severe responses.

**Table 1.** Summary of qualitative and quantitative responses of AFR to dam construction after 99 and 360 years, expressed as a function of pre-dam river dynamics. Rivers with high pre-dam dynamics (Rivers 1 to 4; i.e., single-thread rivers with greater sediment loads, channel gradients, and migration) experience larger incision and develop greater secondary floodplain width, leading to a hydrological disconnection of the active river and its floodplain and lakes. Rivers with lower pre-dam dynamics (Rivers 5 and 6; i.e., multithread rivers with lower sediment loads, channel gradients, and migration) experience similar effects, but impacts are lessened due to the reduced imbalance between pre- and post-dam sediment supply rates. TSS, mean annual total suspended sediment.

Response to dam construction	High pre-dam dynamics (class 1: Rivers 1 to 4)	Low pre-dam dynamics (class 2: Rivers 5 and 6)
Initial channel gradient [−]	0.0015–0.0017	0.0008–0.0011
Initial TSS	140–150 Mt year^−1^	14 Mt year^−1^
Peak vertical incision	Year 99: 2–5 m	Year 99: ∼3 m
	Year 360: 3–7 m	Year 360: 5–6 m
Range of reduction in lateral floodplain reworking to	Up to year 99: 30–70% of reference case	Up to year 99: 30–50% of reference case
	Up to year 360: 20–70% of reference case	Up to year 360: 30–45% of reference case
Annual sediment removal rate	Years 1–99: 10–17 Mt year^−1^	Years 1–99: ∼6 Mt year^−1^
	Years 100–360: 5–7 Mt year^−1^	Years 100–360: 3–4 Mt years^−1^
Average post-dam channel migration	Years 1–360: 12–18 m year^−1^	Years 1–360: 7–10 m year^−1^
Mean secondary floodplain width	Year 99: up to 10 times channel width	Year 99: up to 5 times channel width
	Year 360: up to 15 times channel width	Year 360: up to 10 times channel width
Maximum reduction in peak flood stage	0.5–1.0 m	0.5 m
Area of floodplain inundation	Reduced significantly for all flows along river	Reduced for low and high flows
Abandoned channel cutoff area	1.5–2.0 times area in reference case	1.5 times area in reference case

However, runs for several simulations with varying sediment trapping rates (*1*, *9*) suggest that substantial erosion is expected to occur within the initial 50 to 100 years after dam construction for all trapping scenarios (Fig. 4A). This effect is strongest in rivers with initially low sediment supply rates and low gradients where erosion persists over 360 years in all dam scenarios (River 6). In class 1 meandering rivers, the degree of sediment trapping controls relaxation times until a new morphodynamic equilibrium is reached, which determines net erosion volumes over centuries (Fig. 4A and Table 1). These findings suggest that, although class 1 rivers are highly affected by high sediment trapping, century-scale net erosion can be moderated by reducing trapping at the dam.

Existing fluvial complex-response models [see (*30*) for a review] do not include such morphodynamically driven coupling between channels and floodplain topography, which is critical to ecological assessments of dam impacts and dam placement. Optimization of dam locations should thus consider not only longitudinal connectivity between river transects (*58*) but also local coupling of the channel-floodplain complex, such as the degree of lateral mobility and presence of water bodies with sensitive lentic ecosystems (*16*). Our results highlight that the pre-dam dynamics of the river (here class 1 or class 2), a function of the original channel-floodplain coupling, are critical when assessing the longer-term impacts of dams in rivers that are laterally unconstrained by engineering works.

## DISCUSSION

Previous studies have documented riverine erosion following dam construction, generating downstream changes in river channel slope, width, and bed sediment composition and leading to reduced sediment transport capacity and a new channel equilibrium (*1*, *31*, *59*). Here, we show that this new equilibrium extends to the floodplain, as well as the channel, and depends strongly on lateral erosion that limits channel incision and floodplain water body formation in dynamic rivers: These processes are often ignored in studies predicting geomorphic response to dam construction over longer spatial and temporal scales (*31*, *34*). Lateral reworking compensates for an imbalance in sediment flux and floodplain deposition, which causes rapid floodplain entrenchment over large distances throughout the century after dam construction (Fig. 2) (*12*, *42*). The additional sediment supplied by lateral erosion reduces channel incision, the latter propagating more slowly or over shorter distances downstream than in engineered, laterally constrained rivers (*60*). Our findings contrast with observations from European and North American rivers, where rapid channel incision was observed immediately downstream of dams but where revetments and levees limited bank erosion and promoted downstream distal bed degradation (*61*–*63*). Our results illustrate that although vertical channel incision is buffered through bank erosion in laterally dynamic rivers (*31*), they are likely to respond to newly constructed dams through creation of a secondary floodplain lowered by several meters (Fig. 2C), with ensuing consequences for hydrology and riparian habitat.

Loss of floodplain area has been linked with altered discharge regimes due to dam construction and operation (*38*, *64*). Our results show that additional dam-induced channel and floodplain incision exacerbates the disconnection between the channel and pre-dam floodplain, causing alterations in flood frequency and extent (Fig. 3). This is similar to observations of incised channel reaches in temperate meandering rivers, which have documented a reduction in water level as well as reduced flood extent during frequent smaller floods (*51*, *57*). This disconnection amplifies the impacts of dams on the timing and magnitude of flood peaks (*1*, *65*), altering the hydroperiod of each wetland component (*56*). The reduced and enhanced flooding observed on the original and secondary floodplains, respectively, indicates that floodplains adjacent to rivers will experience higher flood frequencies. However, distal floodplains beyond the entrenched zone lose their connectivity to the channel and will experience a drop in water-table elevations and reduced, but not removed, flood hazard.

These alterations to the flood pulse and resulting inundation have consequences for riparian habitat and consequently the zonation of endemic species (*9*, *66*), with immediate consequences expected for local communities and ecosystems that are dependent on the natural flood pulse (*12*, *67*). The diversity of floodplain species depends strongly on topographic floodplain diversity and associated inundation and soil moisture regimes, and hence ecosystem changes are likely to occur that could persist over centuries (*32*, *56*, *68*, *69*), with critical or “iconic species” being lost irreversibly (*70*). Despite lower rates of channel migration—the process forming cutoff channels and floodplain water bodies and maintaining their inundation regimes (*44*)—cutoff area increases in the most dynamic rivers under the impacts of dams (Fig. 3E). This is due to the observed decoupling of channel and floodplain that inhibits the overbank supply of sediment to the outer zones of the original floodplain and reduces the infilling of cutoffs. This disconnection, and associated reduced water exchanges, could reduce the quality and oxygenation of cutoff waters and disconnect lentic and lotic fluvial ecosystems, with consequences for fish (*13*) and other migratory aquatic organisms that use these lakes for spawning, feeding, and shelter. The impacts of dams will be especially severe in highly dynamic rivers and are likely to be amplified by the cumulative basin-wide effects of multiple dams (*14*, *17*, *25*).

Our ARF models demonstrate that dam construction induces floodplain entrenchment through vertical and lateral erosion, which can change channel-floodplain morphology and the opportunity for floodplain inundation over centuries. Although sediment supply in our simulations depends on discharge and therefore varies over each hydrograph, our simulations assume constant long-term sediment trapping rates, independent of dam operation strategy and seasonal variability in sediment release (*1*). Flexible dam operations, including intermittent flushing, ecological flows, or sediment management, could help mitigate the observed wide-ranging downstream erosion (*7*, *58*). For scenarios with lower trapping rates (dam scenarios C and D), downstream river migration rates slow down less, and lateral erosion can mitigate the degree of vertical incision (Fig. 4 and figs. S8 and S9). However, this buffering capacity is counteracted by development of wider, although less deep, secondary floodplains. This entrenchment affects the floodplain in two ways: (i) by reducing total floodplain area and (ii) by altering flood frequencies. In River 1 (class 1), around 15% of the flooded area is lost compared with the reference scenario. In River 5 (class 2), it is roughly half of that. However, despite maintaining a substantial area of the floodplain in River 5, alterations in flood frequency are severe with an average negative 8.5% shift of the mean flood frequency and a loss of large areas that are frequently flooded (>85% of years). In River 1, the distribution of flood frequencies is less affected, maintaining flood regimes and inundation periods.

On the basis of these observations, we expect that operation strategies that limit sediment trapping, such as flexible dam operation and sediment flushing, could limit vertical incision rates and loss of floodplains but that lateral erosion will continue to drive floodplain entrenchment owing to the positive feedbacks between sediment transport and migration rate (*52*). Potentially, large areas of the floodplain can be maintained if sediment supply at the dam remains above ∼40% of the initial total sediment flux, albeit at altered or lower frequencies (Figs. 3 and 4).

Although we have used the characteristics of Andean foreland basins to illustrate the utility of our model-based effort to floodplain environmental assessment, this approach is applicable in other regions with dynamic sediment-rich rivers facing alteration of their sediment and water regimes. Using these models, adaptive management strategies—for example, sediment flushing, fish and sediment bypasses, run-of-the-river dams, and instream turbines—should thus consider the pre-dam hydrological and morphodynamic characteristics of rivers when seeking to mitigate dam-induced changes (*4*, *71*).

## MATERIALS AND METHODS

To assess the long-term geomorphic and hydrological response of laterally dynamic rivers to upstream dam construction, we applied the River Dynamics Simulator over centennial timescales. We compared the morphodynamic characteristics of our exploratory river models to observations to quantify their applicability and simulated their response under varying discharge and sediment supply scenarios.

### The River Dynamics Simulator (eRiDynaS)

We applied the numerical model eRiDynaS, which is a two-dimensional, depth-averaged River Dynamics Simulator that represents water flow, multifraction sediment transport, bank erosion, and floodplain processes and is described in (*41*, *42*). eRiDynaS applies physically-based conservation principles and empirical process relations to simulate the evolution of rivers with diverse morphologies. More specifically, the model solves the depth-averaged shallow-water equations using a solver that is based on inertia, neglects momentum transport [the IF-solver in (*42*)], and calculates bed load and suspended sediment transport through capacity-based and advection-diffusion transport laws, bed level changes using mass conservation, bank erosion as a function of local flow velocity and bank erodibility, and floodplain friction resulting from dynamic vegetation, the latter depending on rules for colonization, growth, and mortality. The main difference between the two river classes emerges from varying initial channel gradients and resulting equilibrium sediment transport rates that control channel migration and vertical incision (see Table 1 and table S1). Two simulations in class 1 rivers considered rivers with lower bank erodibility. This contributed to differences in river morphology but did not substantially affect channel migration rates (table S1).

Simulations start with a straight initial channel of constant width and depth, bordered by a flat floodplain with a uniform downstream slope (see the Supplementary Materials for details). This initial system evolves into a self-formed channel and floodplain with morphology controlled by imposed flood hydrographs and sediment supply. Model grid cells are classified as channel (unvegetated) or floodplain (partially vegetated), with dynamic transitions between these states during simulations (e.g., due to bank erosion or vegetation colonization) (see the Supplementary Materials for more details on the process representations).

### Parameterizing ARFs

The model grid is 106 km by 16 km with 100-m-wide square grid cells. We first calibrated our six river models by comparing them to planform and morphodynamic data from several Andean rivers (*44*). During calibration, we systematically varied the channel slope, upstream sediment supply, and bank erodibility to reproduce six meandering rivers that reproduce channel dimensions, sediment transport, and channel migration rates similar to single- and multithread rivers in the Upper Amazon Basin (see fig. S1 and table S1). Since channel migration and sediment transport rates are the main processes driving morphological change and river planform, our models were determined as appropriate to assess morphodynamic responses to dams.

Andean river discharges are primarily rainfall driven, resulting in annual unimodal hydrographs. Model scenarios incorporated 150 floods, upscaled geomorphically using a morphological acceleration factor of nine (*72*). This factor, chosen to balance long-term channel change simulation with model stability, equates one flood to 9 years of morphological evolution—a common approach in exploratory morphodynamic modeling.

### Imposing dams in the models

Dam scenarios were simulated by altering discharge hydrographs and sediment supply rating curves at the inlet boundary (see fig. S4). Flood peaks were reduced by 30% and baseflow increased by 15%. Sediment supply *Q*_*s*_ was adjusted to 10% for bedload and to 20% for suspended load of the respective reference value by modifying *a* in the rating curve as a function of discharge *Q* and the two variables *a* and *b* as

$$Q_{S}=a*Q^{b}$$ (1)

Since sediment flux depends nonlinearly on discharge, these changes resulted in a total sediment flux reduction of 90 to 94% under dam scenarios. Many planned Amazonian dam sites are located close to the mountain front where confined gravel-bed rivers transition to freely migrating lowland sand-bed systems. We simulate the geomorphic evolution of these lowland AFR systems.

In addition to the dam scenarios presented herein, we conducted several sensitivity simulations with varying hydrographs and rating curves in rivers 1 and 5 to assess the sensitivity of our results to the dam settings (table S2).

### Comparison with observational data

We used Landsat data to extract river channels in the Beni and Ucayali Rivers for image composites of the years 1987 and 2023 following the method described in (*73*) to compare with the modeled rivers from the last two simulated flood hydrographs (equivalent simulated time between these two time steps is 36 years). We used the rivMap toolbox [in MATLAB (*18*)] to extract centerlines and river channel length and to calculate sinuosity. We assess the model error by calculating the mean error for a channel width-depth ratio, sinuosity, and migration rate between the modeled rivers and observations (*44*) by subtracting mean values of each for the modeled rivers and the Beni and Ucayali Rivers and normalize by the observations (see Fig. 1).

### Model analyses

We extracted and analyzed all model results using Python version 3.11. First, grid cells were classified into channel and floodplain based on the definitions implemented by the model: eRiDynaS defines floodplain cells as those that could sustain vegetation cover because inundation duration does not exceed a threshold period (see SM p. 5 for details on floodplain-channel conversion). We then extracted bed elevations, water levels, and sediment transport rates for channel and floodplain cells. Since grid cells change between channel and floodplain throughout the simulation, we extracted all parameters during both high and low flow of each flood and averaged the results over the second half of the simulation (years 180 to 360).

To assess entrenchment due to dams, we divide all floodplain grid cells into original and secondary floodplain for high and low flows after dam construction. At each time step, we define a grid cell as secondary floodplain if the grid cell was converted from channel to floodplain according to the model. This rule is based on our observation that channel incision and migration are the predominant mechanisms in driving entrenchment and that reworked floodplain cells after dam construction become part of the entrenched floodplain. Sensitivity analyses showed that this rule is effective in extracting the development of entrenchment (defined as mean bed elevations substantially lower than the original floodplain) through time.

To separate cutoff channels from the active channel, grid cells were defined as wet when water depths exceeded 0.1 m, with wet cells that experienced flow velocities > 0.2 m/s classified as channel. The remaining unvegetated wet cells were classified as potential cutoff cells. Cutoffs represent abandoned channels and were therefore extracted as wet cell complexes that comprise at least two connecting cells that do not border the channel and with an elevation below 80% of the median floodplain elevation. Wet cells that did not fulfill these requirements were defined as small floodplain lakes and were not analyzed systematically. Thresholds defining cutoffs were derived from extensive testing to find a generic methodology that could be applied to all rivers and scenarios (fig. S7).

To compute changes in flood frequency due to dams, we counted the number of times each cell was flooded at peak flow for years 180 to 360. We then divided all counts into bins of 5% of all floods and counted the number of cells for each bin to create a histogram for the two classes “original floodplain” and “secondary floodplain.” In the reference (no-dam) scenario, all cells are the original floodplain that is named herein “reference scenario.” Last, we calculated the total area for each bin based on the grid cell size of 10,000 m^2^. For assessing statistical differences between the reference and dam scenarios, we applied a Kolmogorov-Smirnov test that measures the largest difference between the cumulative distributions of two datasets and identifies distributions as significantly different for *P* values < 0.001. The difference in flooded area (Δ*A*) identifies the area of lost floodplain after dam construction compared to the reference scenario. The difference in the mean of both distributions (Δμ) represents the average change in flood frequency disregarding spread and shape of the distributions. The Wasserstein distance *W* determines differences in variable distributions between the scenarios while considering their shape: *W* indicates how far, on average, each value must shift (in percentage points) for one distribution to become identical to the other. The difference in means shows the average change in flood frequency between scenarios, whereas *W* accounts for differences at all flood-frequency levels, including rare and extreme events.

## Supplementary Materials

### This PDF file includes:

Supplementary Text Tables S1 and S2 Figs. S1 to S10 References
