# Supplementary material for: Dams trigger long-term river-floodplain decoupling in dynamic Andean foreland rivers
Source: Sci Adv. 2026 Aug 28;12(35):eaec8647. doi: 10.1126/sciadv.aec8647 (PMC13524042; doi:10.1126/sciadv.aec8647)
Supplement: Supplementary file 1 [file sciadv.aec8647_sm.pdf]

Science Advances
AAAS

Supplementary Materials for

Dams trigger long-term river-floodplain decoupling in dynamic Andean foreland rivers

Muriel Z. M. Brückner *et al.*

Corresponding author: Muriel Z. M. Brückner, mbruckner@lsu.edu

Sci. Adv. **12**, eaec8647 (2026)
DOI: 10.1126/sciadv.aec8647

This PDF file includes:

Supplementary Text
Tables S1 and S2
Figs. S1 to S10
References

Supplementary Text

Supplementary Materials & Methods

Overview of the River Dynamics Simulator

eRiDynaS is a two-dimensional (grid-based, depth-averaged) River Dynamics Simulator that incorporates equations representing water flow, multi-fraction sediment transport, bank erosion and floodplain processes(*41, 42*). Initial channel and floodplain morphology (e.g., river slope, width and depth) and grain size characteristics (for bedload and suspended load) define a channel-floodplain system with a simple initial configuration. The river system then evolves during the simulation to develop a self-formed channel and floodplain with morphology that is a product of the imposed model boundary conditions and process representation. Each grid cell in the domain is classed as either channel (unvegetated) or floodplain (partially vegetated). Cells can be converted between these two classes through a range of mechanisms as a simulation proceeds. We summarize here the main process equations, for more detailed formulations the reader is referred to (*41*).

The hydrodynamics are based on the inertial formulation of the shallow water equations presented by (*75*), which allows the model to be implemented using coarser grid cell sizes, with the benefit of reducing computational cost. In (*41*), this approach is compared to the full version of the depth-averaged shallow-water equations showing that the simpler approach can simulate the evolution of meandering channels (and their floodplains) using coarse model grids. Both bedload and suspended load transport are incorporated via a capacity driven approach for coarse sediment fractions and an advection-diffusion approach for fine fractions. Coarse sediment transport rates are based on a generic velocity-based transport power law function of the depth-mean velocity in excess of a grain size dependent threshold:

$$qb_k = f_k f_b \Omega (\epsilon_F V - Vcr_k)^{\beta} \quad \text{(S1)}$$

$$Vcr_k = C\sqrt{R\tau^*_{cr} D_m^{\chi} D_k^{1-\chi}} \quad \text{(S2)}$$

where qb_k is the volumetric sediment transport rate per unit width for the kth size class, f_k is the fraction of size class k in the active bed layer, f_b is the fraction of the grid cell that is unvegetated, Ω is a constant, V is the depth-mean velocity in the grid cell, β is an exponent, ϵ_F is a transport efficiency factor, which can be used to approximate the effect of form drag, Vcr_k is the critical entrainment velocity of sediment in size class k, C is the Chézy coefficient, R is the submerged specific gravity of sediment, τ^*_{cr} is the critical dimensionless shear stress for entrainment, D_m is the geometric mean sediment diameter in the active layer, D_k is the mean sediment diameter in size class k, and χ is a hiding factor (herein $\Omega = 0.001$; $\beta = 3$; $\epsilon_F = 1$; $\tau^*_{cr} = 0.03$; $\chi = 0.8$).

Coarse sediment transport is steered along streamline curvature via a geometric method to account for curvature effects on sediment transport direction. Sediment fluxes in x and y directions are first calculated at as:

$$qbx_k = qb_k \cos\left(\tan^{-1}\left(v_y/v_x\right) - \tan^{-1}(\epsilon_A h/R_C)\right) \quad \text{(S3a)}$$

$$qby_k = qb_k \sin\left(\tan^{-1}(v_y/v_x) - \tan^{-1}(\epsilon_A h/R_C)\right) \tag{S3b}$$

where qbx_k and qby_k are the x and y components of the volumetric transport rate per unit width for the kth size fraction, Vx and Vy are the x and y velocity components, h is the flow depth, R_C is the streamline radius of curvature at the cell center, which is calculated from the local flow field as described in[1], and ϵ_A is a parameter that controls the magnitude of the curvature effect on sediment transport. We use $\epsilon_A = 20$ which produces channels with realistic meander morphology and dynamics when the model is implemented using a coarse resolution mesh, as here (see[1]; Table S1).

Sediment fluxes ($qbx_{f,k}$ and $qby_{f,k}$) at cell faces (f) are then calculated accounting for topographic effects as:

$$qbx_{f,k} = qbx_k\left(1 + S_{Long}\right)_{UP} - qby_k\left(1 + S_{Long}\right)_{LOW}\Gamma_{Tran}S_{Tran} \tag{S4a}$$

$$qby_{f,k} = qby_k\left(1 + S_{Long}\right)_{UP} + qbx_k\left(1 + S_{Long}\right)_{LOW}\Gamma_{Tran}S_{Tran} \tag{S4b}$$

where S_{Long} and S_{Tran} are the longitudinal and transverse bed slopes, subscripts UP and LOW indicate cells on the upstream and lower sides of the face, and Γ_{Tran} is a coefficient that controls the magnitude of lateral sediment deflection by bed topography.

Transverse bed slope effects are included following the principles outlined by (*76*) and others, summarized by (*77*). We used a transverse sediment transport factor of 5.5 in all simulations that was determined via tests, which is of similar orders of magnitude as in other morphodynamic models (see (*78*)) and was shown to reproduce realistic channel depths (Table S1).

Faces in the model domain that separate channel grid cells from floodplain grid cells are treated as riverbanks. At these faces, the transfer of sediment from the floodplain to the channel cell is represented as lateral bank erosion. The bank erosion process involves two stages: (i) calculation of sediment fluxes from the floodplain to the channel, which results in removal of mass from the floodplain cell; and (ii) conversion of the floodplain cell into a channel cell once sufficient sediment has been removed from the floodplain. Bank erosion rates are controlled by bank erodibility defined by an erosion parameter and the near-bank water flow velocity in excess of a critical flow velocity. The erosion parameter is a multiplier of the calculated bank erosion and was used to introduce varying bank erodibility between AFR to generate a range of migration rates (Fig. S1). Bank erosion rates are then calculated as:

$$q_{sk} = f_{k,F}\, \Phi\, (v_c - v_{cr})\, \omega\, \mathrm{H}(1 - \zeta f_{SC,F}) \tag{S5}$$

where qs_k is the bank erosion sediment flux for size class k, $f_{k,F}$ is the fraction of sediment in the active layer of the floodplain cell in size class k, $f_{SC,F}$ is the fraction of silt and clay sized sediment in the river bank as a whole, V_c is the depth-mean velocity in the bank-adjacent channel cell, V_{cr} is the critical velocity for bank material entrainment set to 0.5 m s^{-1}, H is the bank

height, ω is set to 3, Θ is the bank erodibility, which can be a function of floodplain age but is here a constant (see Table S1), and ζ is an empirical constant here set to 1.

Coarse sediment that is eroded from the floodplain cell is deposited in the adjacent channel cell. In contrast, fine sediment (modelled using a supply-limited approach) enters the water column in the channel cell and then transported using the advection-diffusion equation. To parameterize the different bank erosion in the two classes of rivers, bank erodibility Θ is set in rivers of Class 2 to 10% of Class 1 (Table S1). The sediment rating curves at the inlet were determined such that an initial dynamic equilibrium between channel sediment transport rates, erosion and deposition could be obtained for each river based on their grain sizes, bank erodibility, and initial basin and channel slopes (following equation 1).

Floodplain cells are assumed to contain vegetation, the age of which increases progressively from the point in time when the cell was first converted to floodplain, up to a maximum age value of 900 days morphodynamic time. The floodplain Chézy coefficient and the fraction of the cell that is vegetated are assumed to change linearly as a function of the floodplain surface age, between minimum and maximum values of 25 $m^{1/2}/s$ and 10 $m^{1/2}/s$, respectively. The bare surface fraction, that is used in bedload transport calculations, is one minus the vegetated fraction. Curvature corrections to the sediment transport direction are not applied in floodplain cells. The conversion of channel cells to floodplain is assumed to occur as a result of the onset of vegetation colonization. Cells are assumed to be colonized if the time since the cell was last inundated by more than a threshold (here 15 cm) exceeds the time required for colonization (here set to be 10% of the flood duration). The short time for vegetation colonization used here is chosen to reflect tropical conditions in which vegetation colonizes rapidly.

Model grid cells may be converted from floodplain to channel as a result of several mechanisms, including bank erosion (see above) or vegetation die-off. Die-off occurs through burial by sediment (where the floodplain surface experiences aggradation larger than a threshold (here 1m)), vegetation uprooting (where the floodplain flow velocity exceeds a critical velocity for vegetation scour (here 2.5 m/s)) or mortality due to inundation (if the floodplain surface is inundated by water deeper than 40 cm for a time greater than a threshold duration (70% of the flood duration)). All values were based on typical ranges for Amazonian várzea vegetation (*7*) and are the same in all simulations.

River model parameterization and comparison with Andean foreland rivers

We used Andean foreland rivers (AFR) as inspiration for our six simulated rivers and validated their hydro-geomorphic parameters by comparing them to the characteristics of the Beni and Ucayali rivers. Table S1 presents key parameters of the six modelled rivers at the start of the simulation (initial parameters: channel slope, grain size ranges, bank erodibility factor, water discharge, and sediment fluxes), and the evolving channel parameters after the 450-year spin-up interval (mean channel width, sinuosity, and mean water depth). Migration rate is computed by the sum of total reworked area over the 360 years of reference simulation divided by the centerline length. The presented values are representative of the general river geomorphology without dam construction and are compared to published data for the Beni and Ucayali Rivers.

In general, AFR are white-water, sand-bed rivers meaning that they transport large amounts of sediment, with c. 10% of the total sand transport being transported as bed load (*48*, *79*). The rivers modelled herein have relative bedload transport rates between 2-17% of their total sediment load. Our models also reproduce the presence of a fan at the inlet (see maps in Fig. 10), which is representative of the gravel-sand transition observed in many AFR (*21,80–81*). With distance from the fan, sandy meandering planforms evolve with channel sinuosities <2.3, as reproduced in our six modelled rivers (Table S1). Sinuosity was calculated by dividing total centerline length by valley length during low flow conditions in year 360. We extracted channel width by dividing the total channel area at that time-step by the centerline length. Thus, if present, multiple channels contribute to the total channel width. Mean water depth was defined as the 99th-percentile of the difference between the mean water level in the channel for inbank flow, as an approximation of bankfull conditions, and the mean channel bed elevation.

Table S1. *Hydrogeomorphic parameters of the modelled AFR compared with the Beni and Ucayali Rivers. River parameters for the reference scenarios compared with the Beni River (17, 46-47) and the Ucayali River (21, 46-47, 81), showing good agreement between the natural and simulated geomorphic parameters. Q: discharge range, TBS: mean annual bedload sediment flux, TSS: mean annual suspended sediment flux, D: grain size diameter. Initial parameters were determined at the start of the simulation, channel parameters evolve from the processes in the model.*

**Initial parameters**	**River 1**	**River 2**	**River 3**	**River 4**	**River 5**	**River 6**	**Beni River**	**Ucayali River**
Discharge [m³/s]	2,000-12,000	2,000-12,000	2,000-12,000	2,000-12,000	2,000-12,000	2,000-12,000	2,300-8,920	4,000-13,000
Initial channel gradient [-]	0.00017	0.00015	0.00017	0.00015	0.00011	0.00008	0.00015	0.00010
TSS [Mt yr⁻¹]	140	140	149	149	14	14	212	205
TBS [Mt yr⁻¹]	2.7	2.7	2.8	2.6	2.7	2.7	-	-
Grain size range [mm]	0.032-64	0.032-90	0.032-64	0.032-90	0.032-64	0.032-90	0.007-57	0.15-0.4
Bank erosion factor ϕ[-]	0.0005	0.0005	0.00005	0.00005	0.0005	0.0005	-	-
**Channel parameters**								
Mean channel width [m]	570	510	560	480	670	720	410	600
Sinuosity [-]	1.78	1.96	1.98	2.49	1.87	1.85	2.12	2.11
Bankfull channel depth [m]	11	11	13	14	11	13	12	10
Mean channel migration rate (all channels) [m yr-1]	35	37	36	33	30	32	30	36

Sediment transport rates and channel migration rates are major morphodynamic parameters that are interlinked with each other and many other geomorphic processes, such as bar formation, bank erosion, levee breaching, and floodplain reworking (*46-48, 55*). To assess the channel dynamics in our modelled rivers, we compare relative channel migration rates to average channel width and total suspended sediment fluxes with data from different reaches in five AFR (*46*). The model predicts migration rates and total suspended sediment fluxes of similar orders of magnitude to those reported in AFR (Fig. S1). Migration rates were calculated as the total area changing from floodplain to channel over each flood divided by the channel length at the end of the simulation, which is representative of the equilibrium channel conditions. Mean transport rates are the averaged integrated values of the sediment supply rating curve at the inlet boundary. The close alignment of the modelled rivers with real AFR shows that the model is able to represent the geomorphic behavior of various AFR, and illustrates the suitability of the model to study their response to damming.
Extraction of the main channel centerline was based on channel flow direction and magnitude, where highest flow velocities directed towards the downstream were defined as the centerline. This approach guarantees that recirculating flow in bends and in-channel bars are excluded from centerline computations. However, this approach results in potential underpredictions of channel length in multi-channel rivers as the main channel is chosen as representative of channel length used in the sinuosity and migration rate computations (Fig. S2).

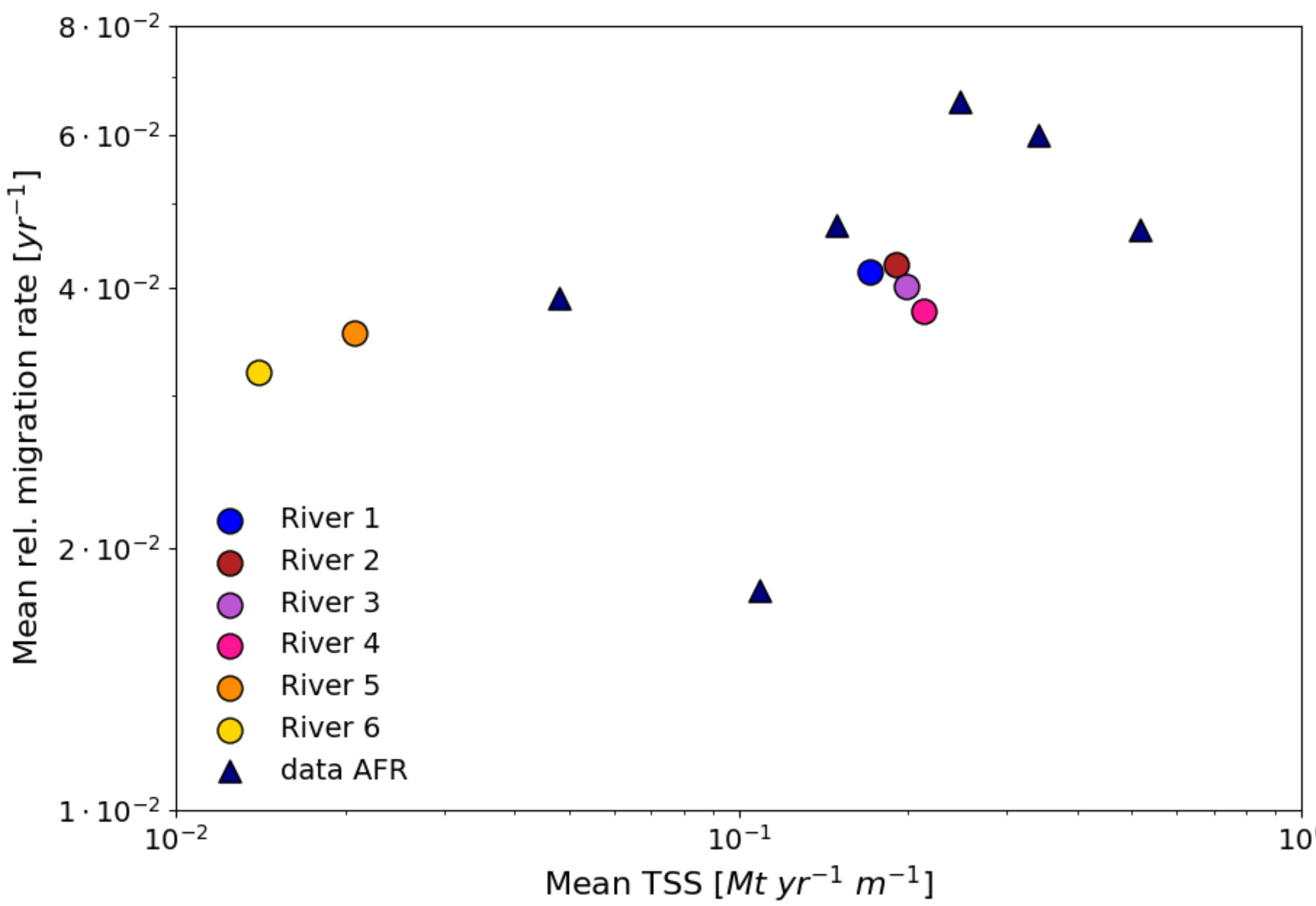

Fig. S1. *Comparison of mean migration rate relative to the average channel width and total suspended sediment flux (TSS) for the reference (no-dam) scenario of the six modelled rivers (colors) with published data from the field (squares). Model results reproduce rivers of similar dynamics and suspended transport rates as reported from the Mamoré, Beni, Ucayali, Madre de Dios, and Huallaga rivers (triangles), which span a wide range of AFR (46).*

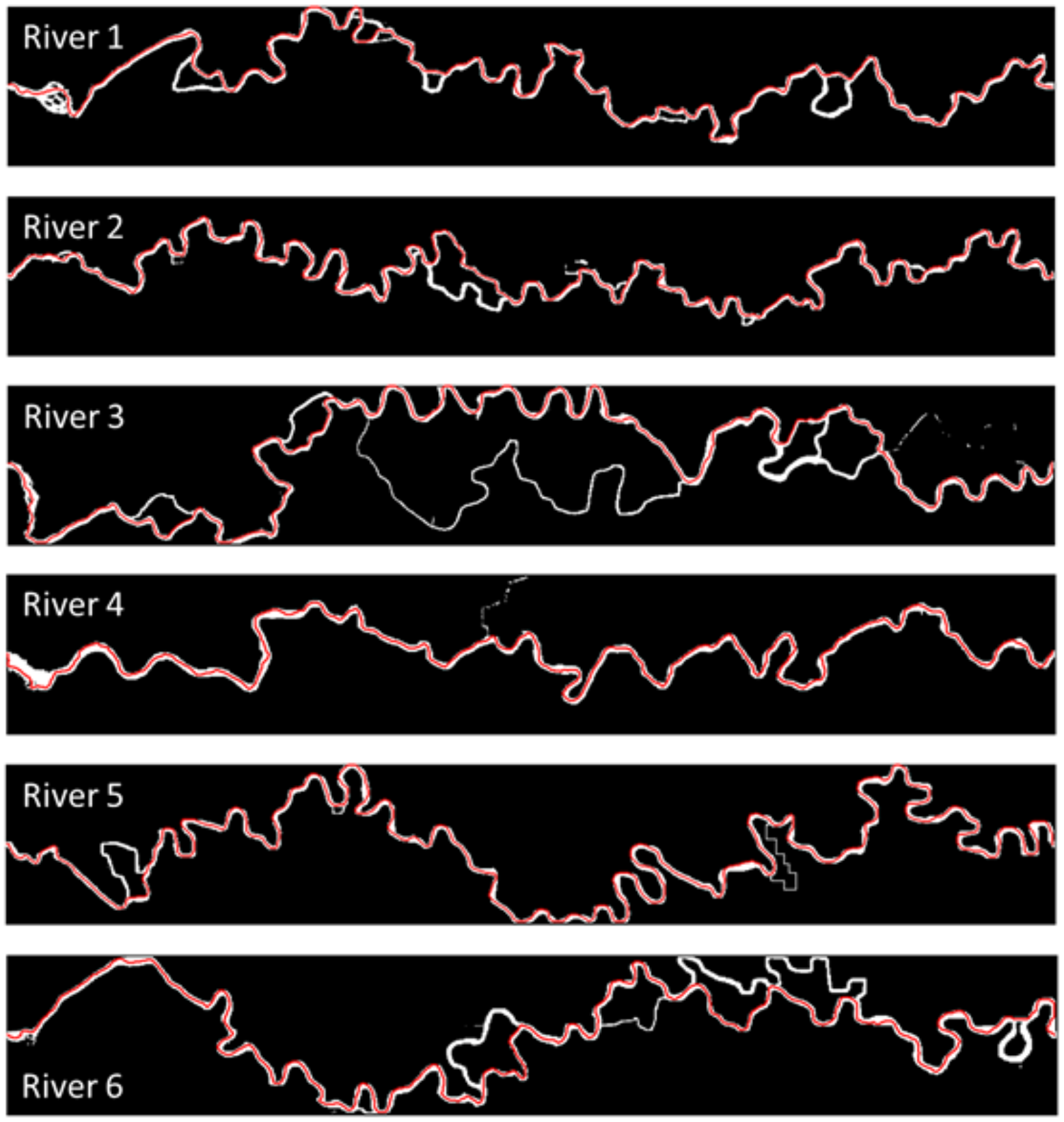

Fig. S2. *Definition of centerline (red lines) in the six modeled reference scenarios (white is channel, black is floodplain). The extracted centerlines were used to compute sinuosity, migration rates, and channel width for the six modelled rivers.*

Parameterization of model scenarios

Water discharges in Andean rivers are driven principally by rainfall, resulting in annual unimodal hydrographs. The model scenarios were driven by 90 floods and upscaled geomorphically using a morphological acceleration factor (*72, 82*) of nine, resulting in one flood representing nine years of morphological change. A factor of nine was selected to enable the simulation of long periods of channel change while maintaining model stability. This type of upscaling is widely applied in exploratory long-term morphodynamic models and has been shown effective for factors up to 500 depending on the application.

In all rivers, the discharge for the references (no-dam) scenarios was defined as a random combination of flood peaks between 8,000-12,000 m^3/s and a baseflow of 2,000 m^3/s based on values reported from the Beni River (*17*) (Fig. S1; Table S1) and a flood duration of 40 days. Total bed load and sediment flux Qs are defined as a power law depending on discharge Q and two variables *a* and *b* (see equation (1) in main article). In Rivers 1-4, *a* is set to $6 \cdot 10^{-7}$ and $2.5 \cdot 10^{-3}$ for bed load and suspended load, respectively. In Rivers 5 and 6 *a* is set to $6 \cdot 10^{-7}$ and $2.5 \cdot 10^{-4}$ for bed load and suspended load, respectively. *b* is set to 1.50 and 1.66 in all rivers for bed load and suspended load, respectively. These values were derived iteratively from extensive testing by adjusting the initial channel (and floodplain) slopes for each river to produce a 'quasi-

equilibrium' morphology at the end of the 450-year spin-up period (Fig. S3) – e.g., the river morphology evolved to a dynamic equilibrium condition that is able to convey the imposed bedload and suspended load supply without experiencing significant net aggradation or degradation between floods.

Dam scenarios were computed by manipulating the discharge hydrographs and sediment supply rating curves at the inlet boundary. Fig. S4 depicts 200 floods for the reference (no-dam) and dam scenario as an example. The Dam A scenario was defined by a reduction of flood peaks by 30% and a baseflow increase by 15%. Sediment supply was mediated by adjusting α in the rating curve to 10% for bed load and 20% for suspended load of the reference value, respectively (*1,83*). Because sediment supply is a function of varying discharge defined by the hydrograph, the reduction in sediment flux is non-linear, resulting in a total reduction in sediment flux by 91-92% in the Dam A scenario.

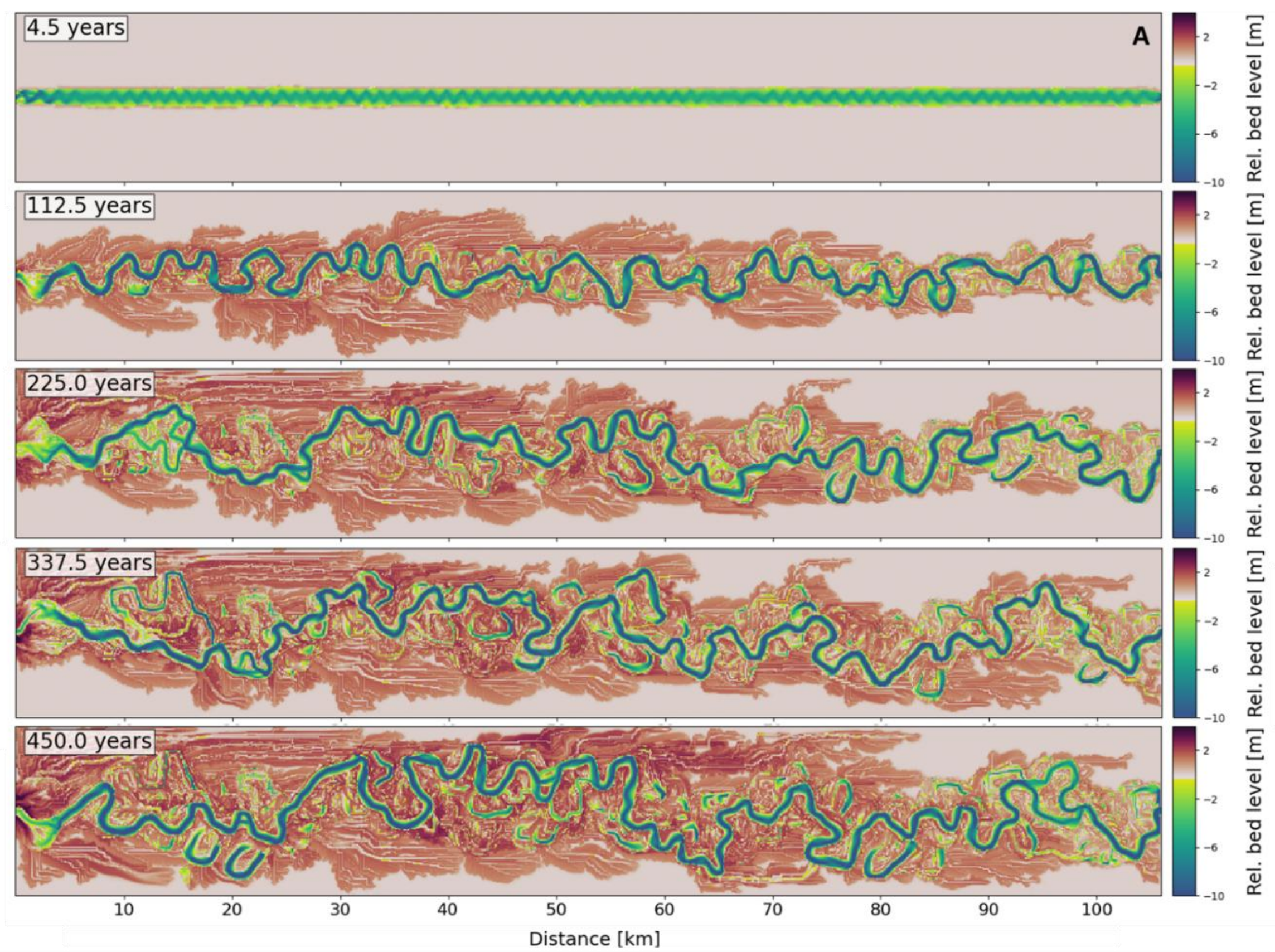

Fig. S3. *Elevation maps for five time-steps during the spin-up period (years 0-450 pre-dam) illustrating the temporal evolution of the morphology of River 1 as an example.*

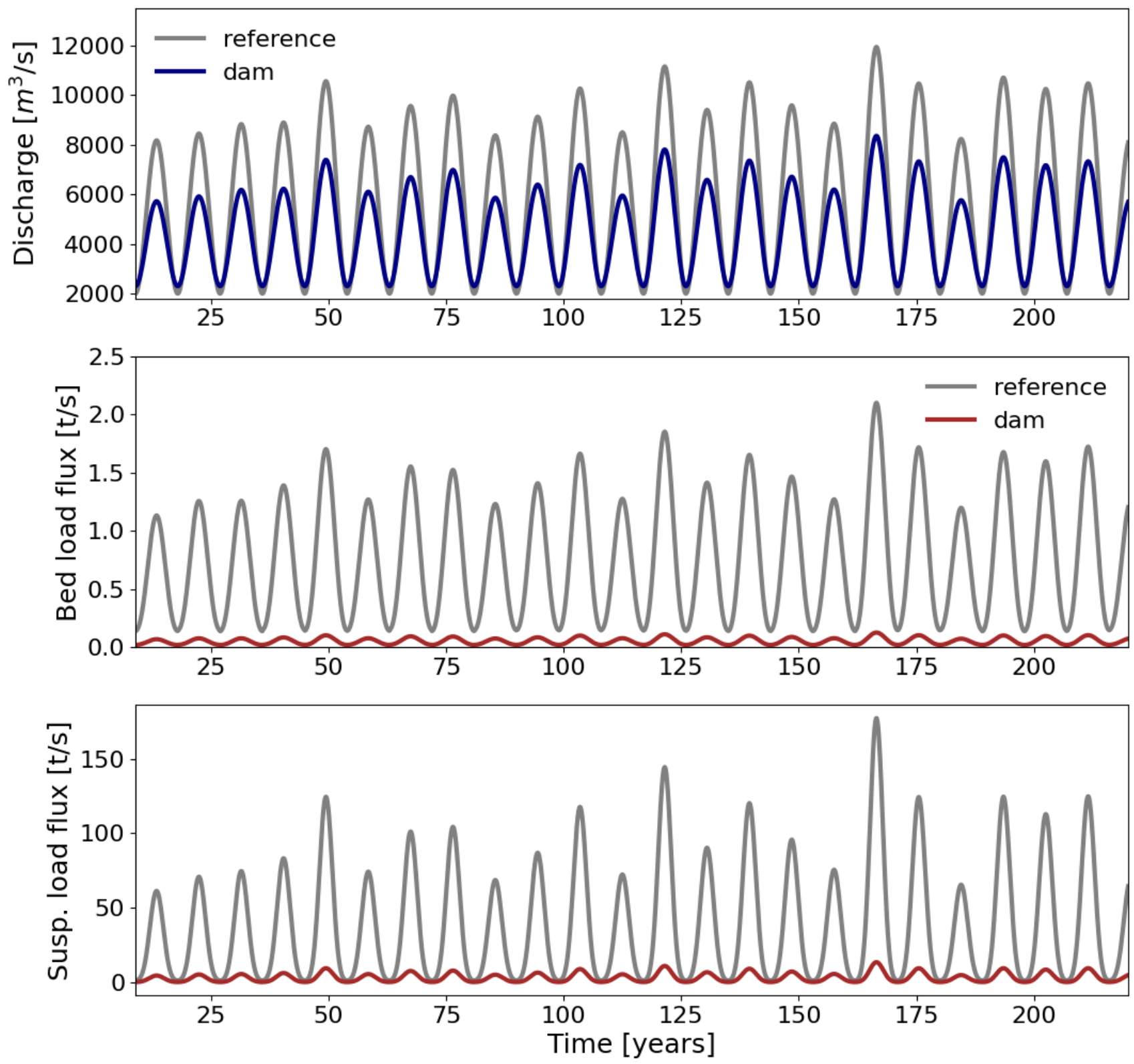

Fig. S4. *Water and sediment rating curves at the inlet for the reference (no dam) and the Dam A (dam) scenario of River 5 as an example. Each randomly generated flood peak represents 9 years of morphological time. In the dam scenario, sediment supply is reduced calculated by the power function as described above (also see equation 1 in main article).*

Figure S5 illustrates how the average annual erosion rate in the model domain declines over time after dam construction. Immediately after dam construction (years 1-50), erosion rates are high in all rivers but over time reduce and converge towards mean erosion rates of 5 Mt/yr. Variable hydrographs cause variable erosion rates between years. Class 2 river rates are lower than Class 1 river rates but also decline more gradually. Class 1 rivers show large initial erosion and a stronger adjustment of erosion rates over time with a steeper slope. The bar plots show that mean net erosion over the first century after dam construction is 2-3 times higher than over the remaining 261 years.

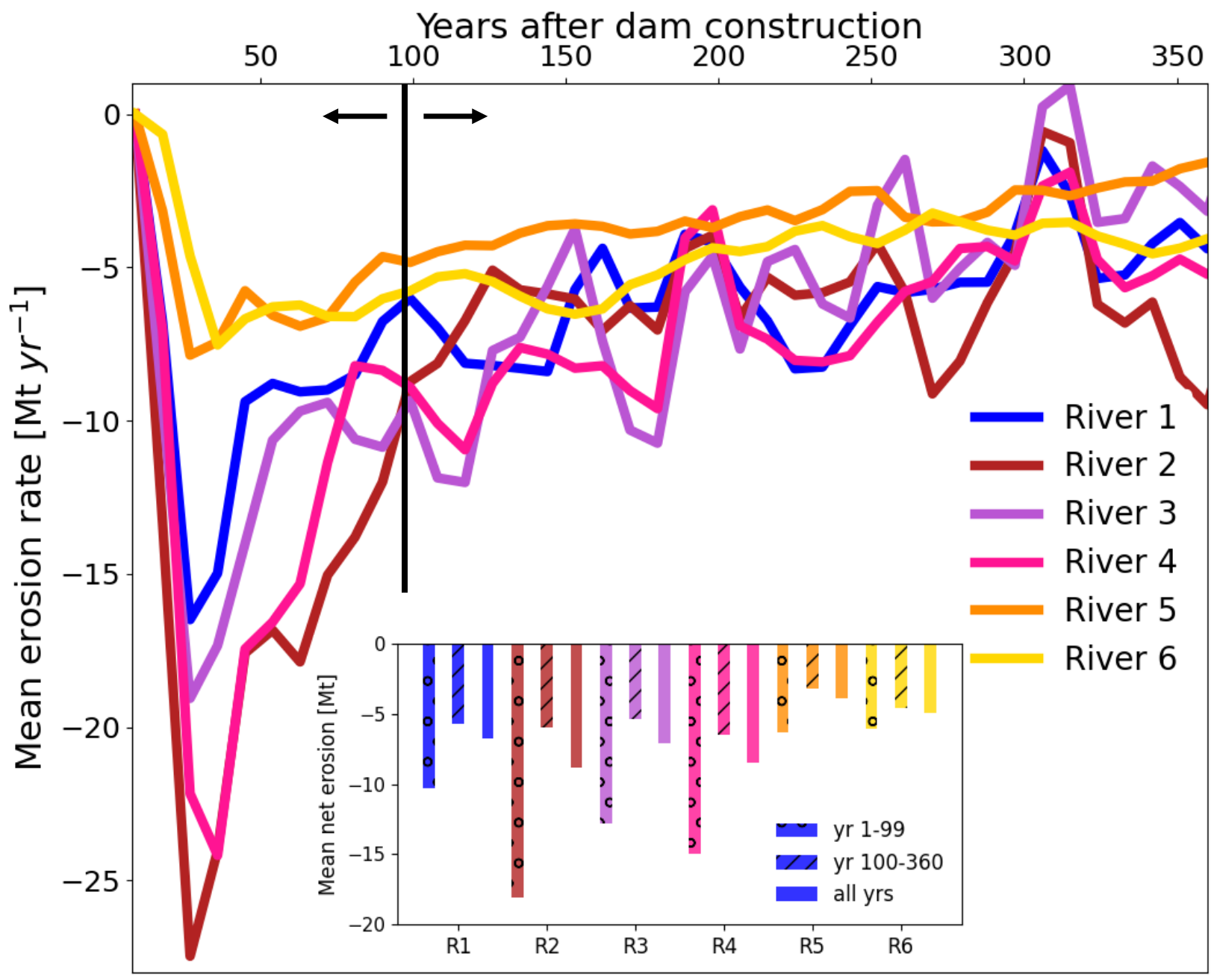

Fig. S5. Erosion rate of the dam scenarios in all rivers compared with mean net erosion in year 1-99 and 100-360. *Mean annual erosion rate over the simulation time computed as the net bed level change between consecutive floods and averaged over 2 floods (lines). The vertical black line marks year 99 marking a strong average decline in erosion rate. Inset bar plot shows the mean net erosion during the first 99 years, the remaining 261 years and for all years combined, showing that erosion rates are largest in the first century after dam construction. Note that flood magnitudes are different between years, leading to annual variability in net erosion.*

<u>Additional model results showing adaptation of AFR to dams</u>

This section presents additional results to the results presented in the publication for the simulations of Rivers 1, 2, 3, and 5. Stage-discharge relationships illustrate how mean water levels drop for after dam closure , especially in areas close to the dam where incision is largest (Fig. S6A and 2D). This drop in water level has severe consequences for floodplain inundation (Fig. S6B): large areas of low to intermediate flood frequencies (1-40% of years) are lost. Water levels rarely reach the original floodplain level, especially in highly eroded rivers (e.g., Rivers 2 and 3), and flood inundation is largely restricted to the secondary floodplain. In less eroded rivers (e.g., River 5), this disconnection is less pronounced, but large areas of inundation at intermediate flood frequencies (30-60% of years) are increased while regularly flooded (70-99% of years) and intermittently flooded areas (< 25%) are reduced.

Lower water levels caused by the combined effects of post-dam discharge regimes and the erosion of the channels and floodplain lead to a disconnection of floodplain water bodies (cutoff channels) located on the original floodplain from the active channel. This produces a greater total area of floodplain lakes in dammed compared to no-dam simulations (Fig. S6C) as sediment supply is stopped and lake infilling ceases.

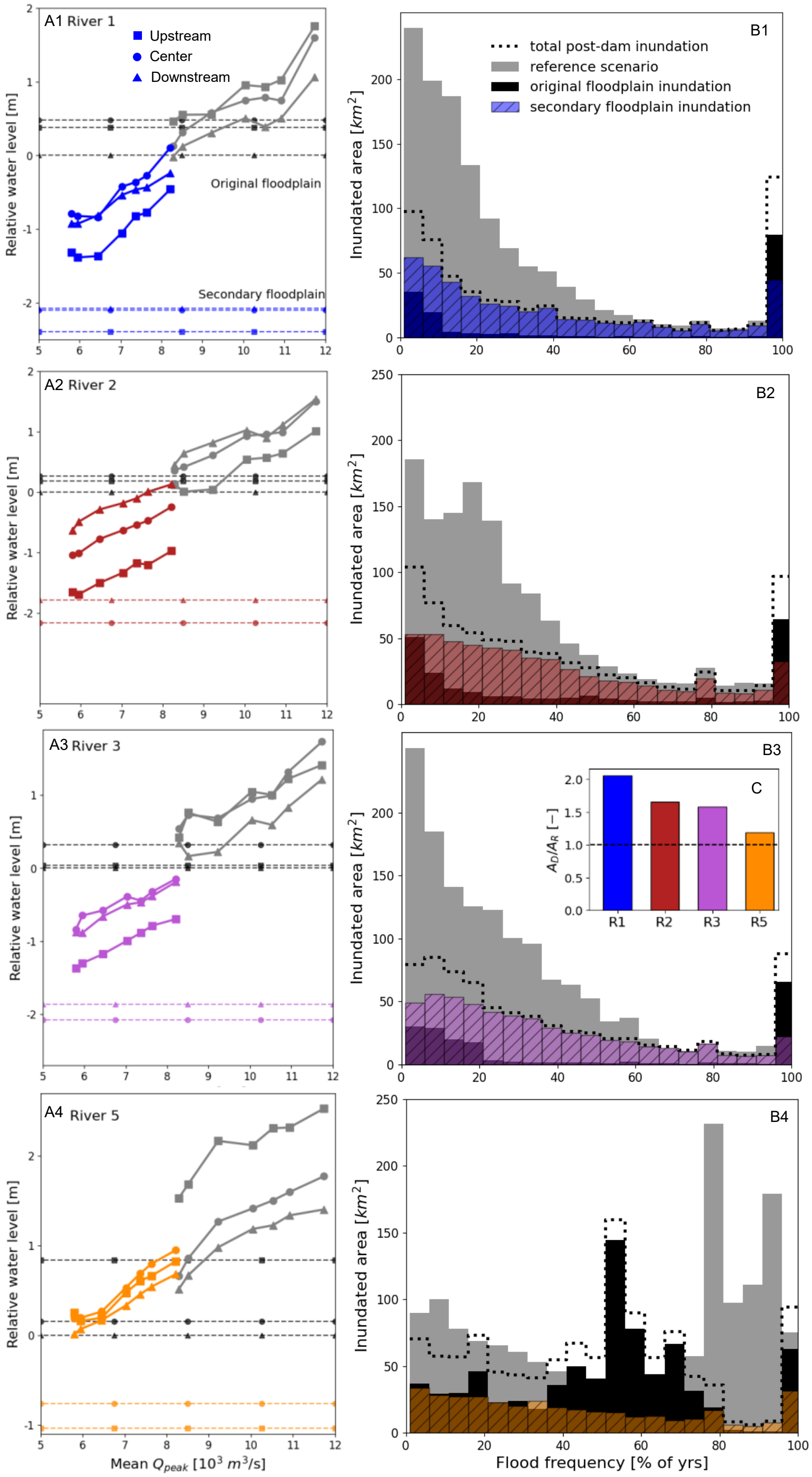

Fig. S6. *Hydrological changes following dam closure for Rivers 1-3 and 5. (A1-4) Relationship between averaged peak flood discharge and relative water level in the reference (no-dam, gray lines) and dam scenarios (colored lines). Data for dam scenarios are averaged over years 180-*

360 (solid lines) and each region (upstream, central and downstream) of the model domain (symbols). Dashed lines represent the mean floodplain elevation of the original floodplain (black lines) and secondary floodplain (colored lines) in each region. Water level is normalized by the mean secondary floodplain elevation in the downstream reach. (B1-4) Area of floodplain inundation plotted as a function of flood frequency averaged over years 180-360 for all rivers (colors as in A). Gray bars indicate inundation in the no-dam scenario. Dotted line represents total floodplain inundation in dam scenarios, which is the sum of the inundation of the original (black bars) and secondary (colored bars) floodplains. (C) Ratio of unfilled cutoff area for each river (1, 2, 3, and 5) at the end of the dam scenario, AD, and the no-dam scenario, AR, (colors as in A).

Cut-off channels are abandoned river channels that result from the river shortening its course during channel migration. To extract abandoned cutoff area from the simulation results, we define cut-offs as floodplain cell complexes (i.e, at least two connecting cells (total area of 2 ha)) with elevations below 80% of the median floodplain elevation that do not border the channel (Fig. S7). These rules were defined through extensive testing to 1) exclude single flooded cells that are inundated due to high local bottom friction, 2) exclude cells immediately adjacent to the channel that have been classified as floodplain by the model due to low flow velocities, and 3) use a generic approach that can extract cut-off area across all rivers and for both the reference and dam scenarios despite varying dynamics and bed elevation distributions. In all rivers (Fig. S7 displays Rivers 1, 5, and 6), cut-off area increases under the impact of dams as compared to the reference scenarios. This increase results from the disconnect between channel and the wider floodplain due to erosion that inhibits 1) sediment transport towards cut-offs needed for infilling and 2) reconnection of older cut-offs to the river by channel migration.

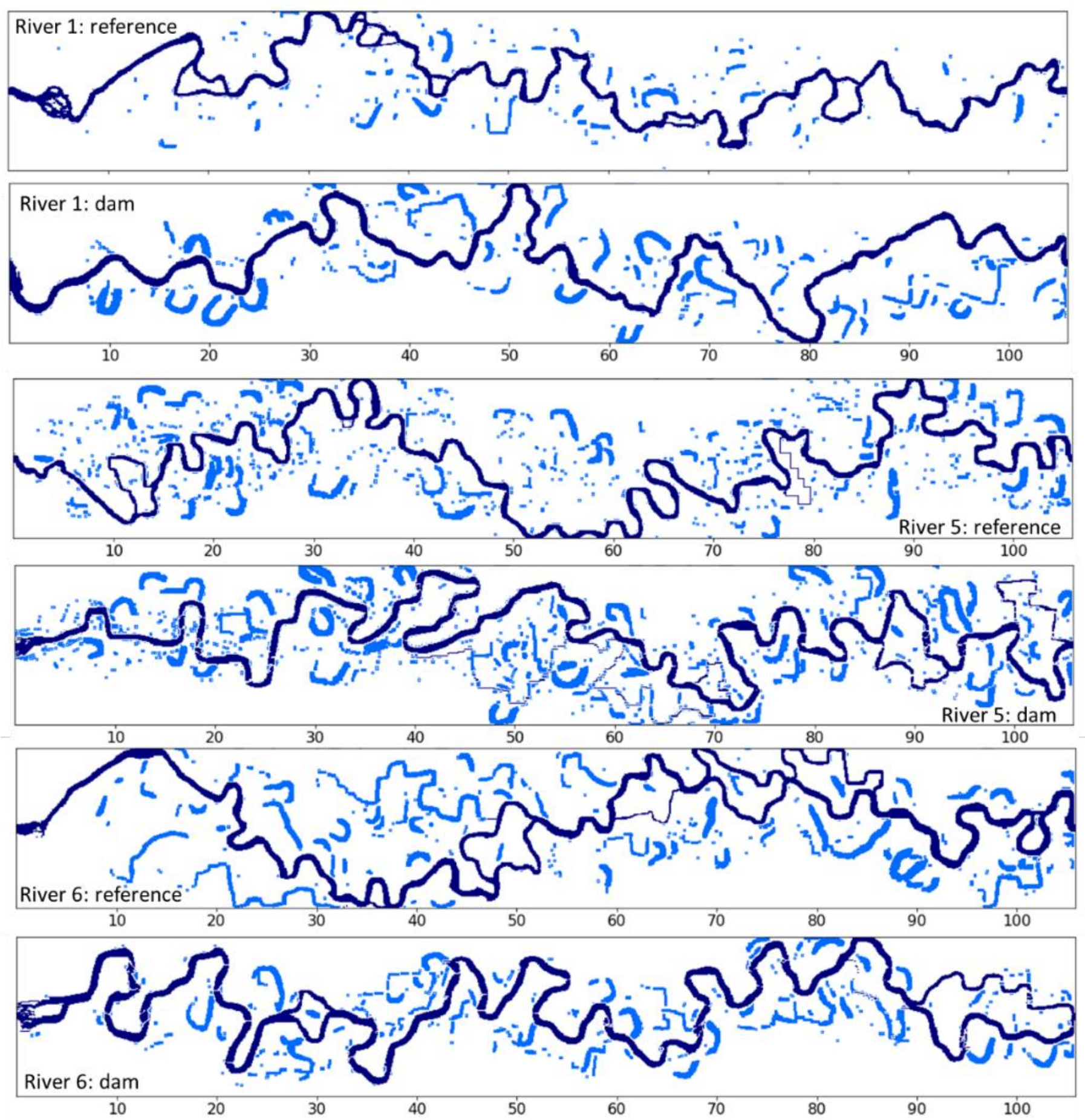

Fig. S7. Maps of active (dark blue) and cut-off channels (pale blue) in three rivers extracted from the simulations. Cut-off area increases in the dam-scenarios compared with the reference (no-dam) scenarios all rivers.

Dam scenarios

To test the effects of varying degrees of damming on the response of AFR, we ran three additional scenarios (Dam B, C, and D) with variable total sediment supply for Class 1 River 1 and Class 2 River 5 (Table S2). The scenarios represent the same discharge regime after dam construction as in Dam scenario A, but varying sediment supply by adjusting the multiplier in the sediment rating curve that leads to sediment supply as presented in Table S2. Note, that the varying rates between River 1 and 5 are a result of the nonlinear relationship between discharge and sediment flux (equation 1 in main article), resulting in disproportionally higher sediment loads at low flows as compared to the no-dam scenario where low flows are smaller.

The results from the sensitivity runs show similar behaviour of Rivers 1 and 5 in all dam scenarios as in the original dam scenario (Dam A): altered flow and sediment regimes below dams induce both vertical channel erosion and lateral floodplain erosion in all scenarios. In all dam scenarios, channel and floodplain incision are highest close to the dam (upstream) and decline downstream (Fig. S8). Although channel incision is of similar magnitude in all dam

scenarios, with reduced sediment trapping (Dams C and D) floodplain incision is reduced, leading to reduced secondary floodplain formation. With increasing sediment transport rates due to reduced trapping, wider but less incised secondary floodplains evolve. As a result, the degree of sediment trapping at the dam does not affect the degree of channel incision but instead controls erosion of the floodplain. However, these findings indicate that the pre-dam conditions of the rivers control the degree of net erosion for varying sediment supply rates at the dam, with the less active river (River 5) showing less variability in erosion between dam scenarios than the Class 1 River 1. In River 1, hydrological disconnection of the main channel from its floodplain is strongest for high sediment trapping at the dam (A, B), but declines with lower trapping (C, D), leading to a total floodplain area loss of 24% and 15% in dam scenario B and C, respectively (Fig. S9). In Class 2 River 5, hydrological disconnection occurs independent of the sediment trapping rates, showing that dams will cause severe changes in hydrological connectivity of these rivers. Although total floodplain area loss is 18% and 11% in dam scenarios B and C, respectively, inundation patterns shift towards lower inundation (Fig. S8): Floodplain entrenchment (Fig. S8) causes a reduction in areas that are frequently flooded while low to intermediate flooding regimes (10-40% of years) increase (Fig. S9).

Table S2.

Sediment supply in the model sensitivity runs, expressed relative to conditions in the reference (no-dam) simulations. Sensitivity runs were only conducted for Rivers 1 (Class 1) and 5 (Class 2). Bed load and suspended load supply were adjusted by reducing *a* in equation 1 leading to the total sediment supply rates below.

Dam scenario	River 1: Total sediment supply [% of reference]	River 5: Total sediment supply [% of reference]
Dam A	8%	9%
Dam B	14%	16%
Dam C	42%	30%
Dam D	54%	40%

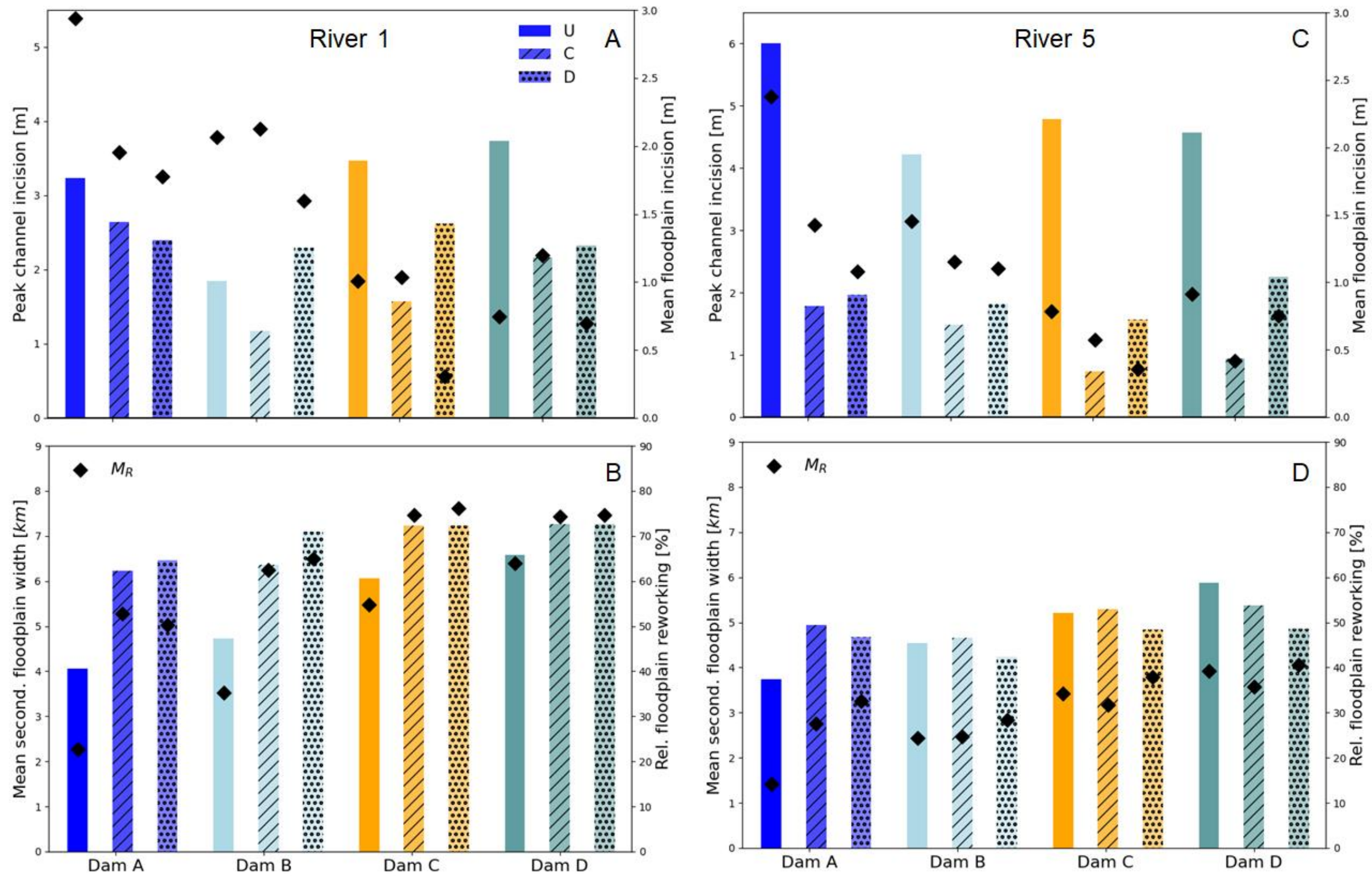

Fig. S8. *Mean channel (histograms) and secondary floodplain (symbols) vertical incision for the four dam scenarios and the upstream, center, and downstream reaches for Rivers 1 and 5 (U,C,D) (A, C). Mean floodplain width (histograms) and rate of floodplain reworking (*M_R*, symbols) for the five dam scenarios and the upstream, center, and downstream reaches for Rivers 1 and 5 (B, D).*

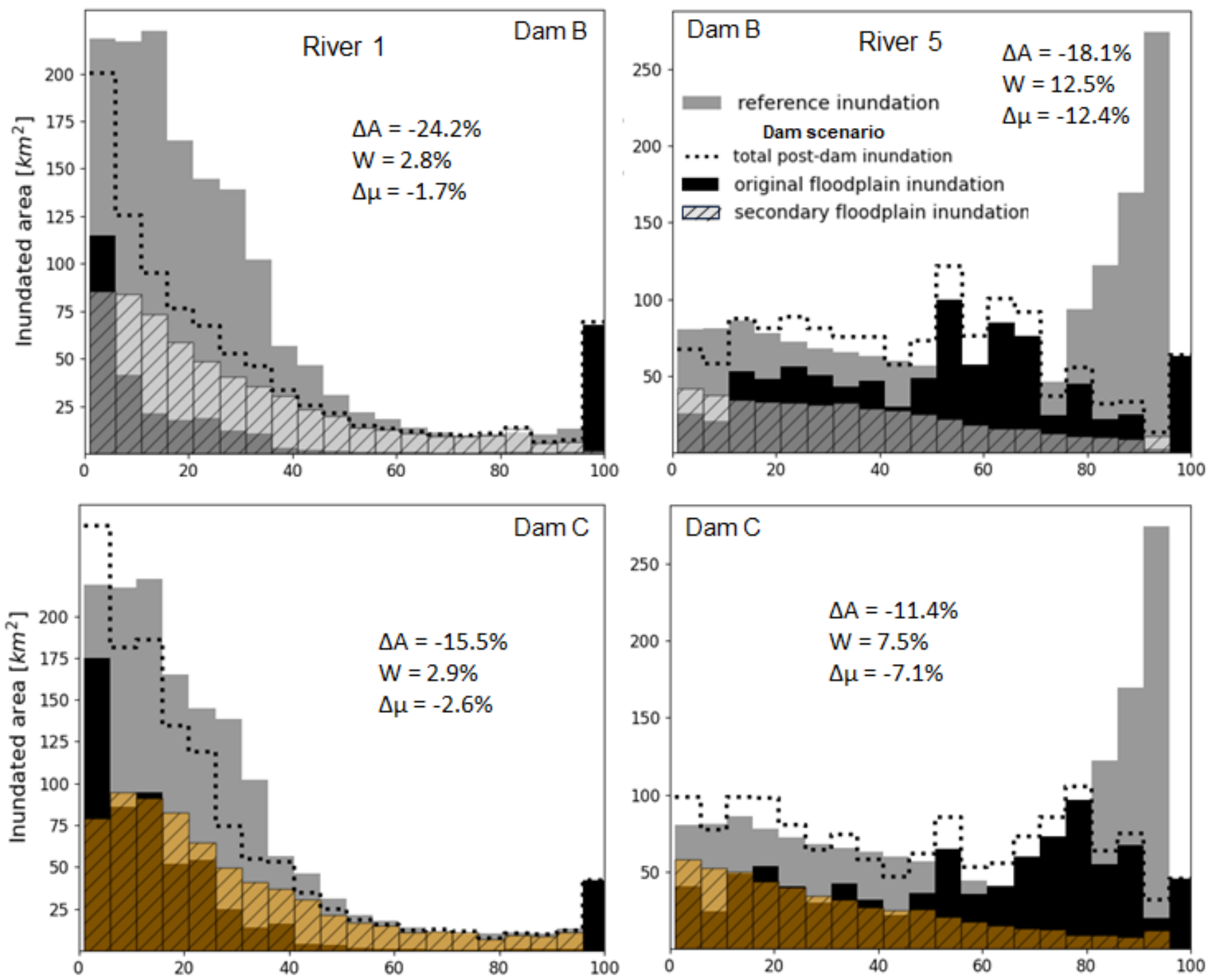

Fig. S9. *Flood frequency histograms comparing the reference and the dam scenarios B and C. Hydrological changes following dam closure for the five dam scenarios in River 1 (Class 1; left) and River 5 (Class 2; right). Area of floodplain inundation plotted as a function of flood frequency (data are averaged over second half of the simulation (year 180-360 after dam closure); bin size = 5 years) for the two rivers and the additional dam scenarios. Gray bars indicate inundation in the reference (no-dam) scenario. Dotted line represents total floodplain inundation in dam scenarios, which is the sum of the inundation of the original (black bars) and secondary (colored bars) floodplains. Kolmogorov-Smirnoff tests showed significant differences ($p << 0.001$) between total post-dam and reference inundation distributions. ΔA represents the difference in flooded area, W Wasserstein distance and Δμ shift in the mean between total post-dam and reference inundation.*

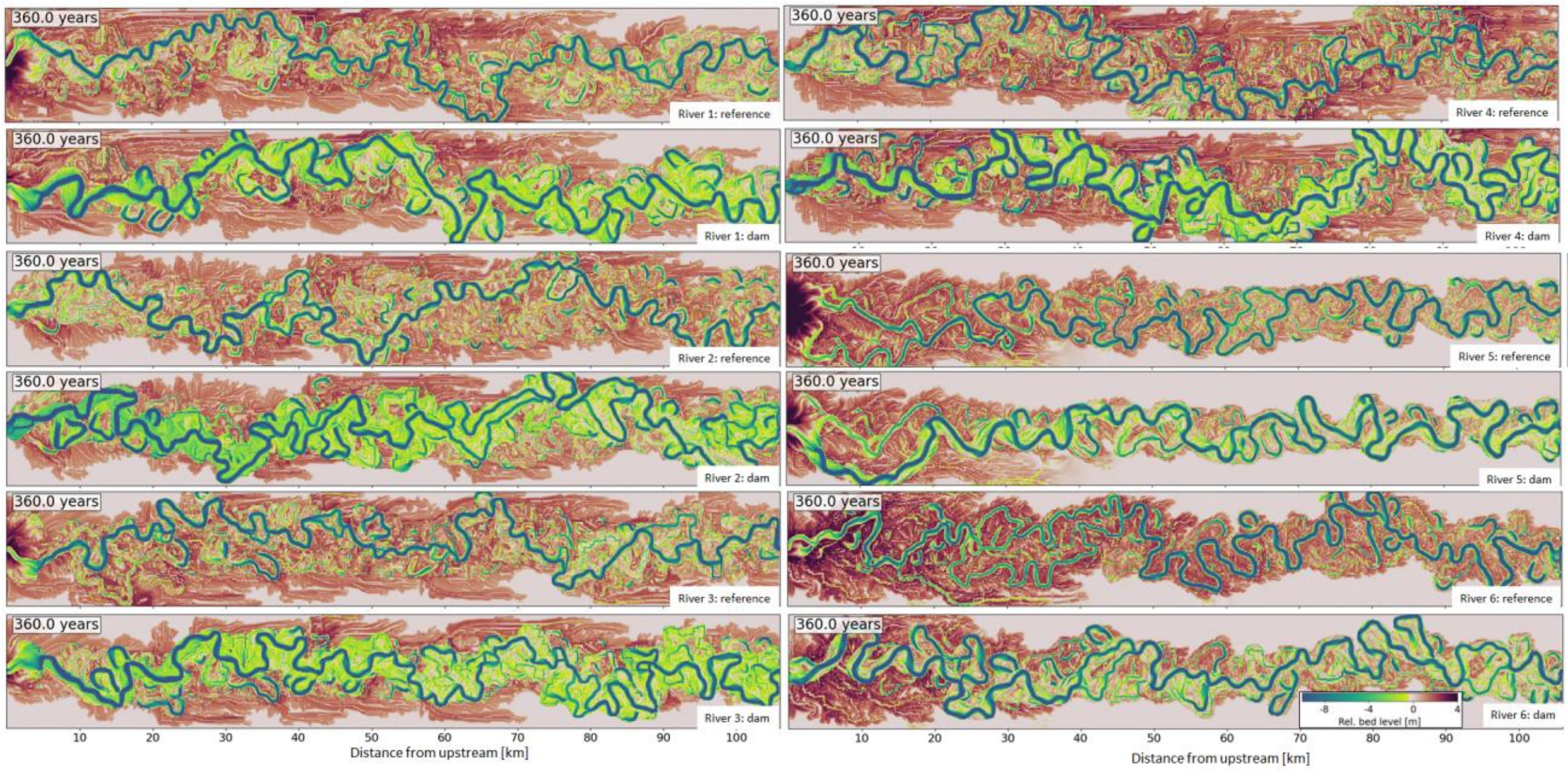

Fig. S10. *Elevation maps of all simulations for the entire modeling domain**.** The elevation maps show the development of a secondary floodplain in all rivers that disconnect the main channel from the floodplain*.

REFERENCES

1. X. Y. Chong, D. Vericat, R. J. Batalla, F. Y. Teo, K. S. P. Lee, C. N. Gibbins, A review of the impacts of dams on the hydromorphology of tropical rivers. *Sci. Total Environ.* **794**, 148686 (2021).

2. C. Zarfl, A. E. Lumsdon, J. Berlekamp, L. Tydecks, K. Tockner, A global boom in hydropower dam construction. *Aquat. Sci.* **77**, 161–170 (2015).

3. Q. Tian, K. H. Xu, Y. P. Wang, C. J. Liang, Dam impacts on seasonality of water and sediment transport in intensively managed river basins: A case study of the Yangtze River. *J. Geophys. Res. Earth Surf.* **127**, e2021JF006573 (2022).

4. E. P. Anderson, C. N. Jenkins, S. Heilpern, J. A. Maldonado-Ocampo, F. M. Carvajal-Vallejos, A. C. Encalada, J. F. Rivadeneira, M. Hidalgo, C. M. Cañas, H. Ortega, N. Salcedo, M. Maldonado, P. A. Tedesco, Fragmentation of Andes-to-Amazon connectivity by hydropower dams. *Sci. Adv.* **4**, eaao1642 (2018).

5. J. Best, Anthropogenic stresses on the world's big rivers. *Nat. Geosci.* **12**, 7–21 (2019).

6. Z. K. Rubin, G. M. Kondolf, P. A. Carling, Anticipated geomorphic impacts from Mekong basin dam construction. *Int. J. River Basin Manag.* **13**, 105–121 (2015).

7. G. M. Kondolf, Y. Gao, G. W. Annandale, G. L. Morris, E. Jiang, J. Zhang, Y. Cao, P. Carling, K. Fu, Q. Guo, R. Hotchkiss, C. Peteuil, T. Sumi, H. Wang, Z. Wang, Z. Wei, B. Wu, C. Wu, C. T. Yang, Sustainable sediment management in reservoirs and regulated rivers: Experiences from five continents. *Earths Future* **2**, 256–280 (2014).

8. H. O. Fagundes, A. S. Fleischmann, F. M. Fan, R. C. D. Paiva, D. C. Buarque, V. A. Siqueira, W. Collischonn, P. Borrelli, Human-induced changes in South American river sediment fluxes from 1984 to 2019. *Water Resour. Res.* **59**, e2023WR034519 (2023).

9. B. R. Forsberg, J. M. Melack, T. Dunne, R. B. Barthem, M. Goulding, R. C. D. Paiva, M. V. Sorribas, U. L. Silva, S. Weisser, The potential impact of new Andean dams on Amazon fluvial ecosystems. *PLOS ONE* **12**, e0182254 (2017).

10. E. Greenberg, V. Ganti, The pace of global river meandering influenced by fluvial sediment supply. *Earth Planet. Sci. Lett.* **634**, 118674 (2024).

11. J. V. Ward, K. Tockner, F. Schiemer, Biodiversity of floodplain river ecosystems: Ecotones and connectivity1. *Regul. Rivers Res. Manage.* **15**, 125–139 (1999).

12. E. M. Latrubesse, F. M. d'Horta, C. C. Ribas, F. Wittmann, J. Zuanon, E. Park, T. Dunne, E. Y. Arima, P. A. Baker, Vulnerability of the biota in riverine and seasonally flooded habitats to damming of Amazonian rivers. *Aquat. Conserv.* **31**, 1136–1149 (2021).

13. Q. Chen, Q. Li, Y. Lin, J. Zhang, J. Xia, J. Ni, S. J. Cooke, J. Best, S. He, T. Feng, Y. Chen, D. Tonina, R. Benjankar, S. Birk, A. S. Fleischmann, H. Yan, L. Tang, River damming impacts on fish habitat and associated conservation measures. *Rev. Geophys.* **61**, e2023RG000819 (2023).

14. K. O. Winemiller, P. B. McIntyre, L. Castello, E. Fluet-Chouinard, T. Giarrizzo, S. Nam, I. G. Baird, W. Darwall, N. K. Lujan, I. Harrison, M. L. J. Stiassny, R. A. M. Silvano, D. B. Fitzgerald, F. M. Pelicice, A. A. Agostinho, L. C. Gomes, J. S. Albert, E. Baran, M. Petrere Jr., C. Zarfl, M. Mulligan, J. P. Sullivan, C. C. Arantes, L. M. Sousa, A. A. Koning, D. J. Hoeinghaus, M. Sabaj, J. G. Lundberg, J. Armbruster, M. L. Thieme, P. Petry, J. Zuanon, G. T. Vilara, J. Snoeks, C. Ou, W. Rainboth, C. S. Pavanelli, A. Akama, A. Soesbergen, L. Sáenz, Balancing hydropower and biodiversity in the Amazon, Congo, and Mekong. *Science* **351**, 128–129 (2016).

15. J. Salo, R. Kalliola, I. Häkkinen, Y. Mäkinen, P. Niemelä, M. Puhakka, P. D. Coley, River dynamics and the diversity of Amazon lowland forest. *Nature* **322**, 254–258 (1986).

16. A. S. Flecker, Q. Shi, R. M. Almeida, H. Angarita, J. M. Gomes-Selman, R. García-Villacorta, S. A. Sethi, S. A. Thomas, N. LeRoy Poff, B. R. Forsberg, S. A. Heilpern, S. K. Hamilton, J. D. Abad, E. P. Anderson, N. Barros, I. Carolina Bernal, R. Bernstein, C. M. Cañas, O. Dangles, A. C. Encalada, A. S. Fleischmann, M. Goulding, J. Higgins, C. Jézéquel, E. I. Larson, P. B. McIntyre, J. M. Melack, M. Montoya, T. Oberdorff, R. Paiva, G. Perez, B. H. Rappazzo, S. Steinschneider, S. Torres, M. Varese, M. Todd Walter, X. Wu, Y. Xue, X. E.

Zapata-Ríos, C. P. Gomes, Reducing adverse impacts of Amazon hydropower expansion. *Science* **375**, 753–760 (2022).

17. E. Gautier, D. Brunstein, P. Vauchel, M. Roulet, O. Fuertes, J. L. Guyot, J. Darozzes, L. Bourrel, Temporal relations between meander deformation, water discharge and sediment fluxes in the floodplain of the Rio Beni (Bolivian Amazonia). *Earth Surf. Process. Landf.* **32**, 230–248 (2007).

18. J. Schwenk, A. Khandelwal, M. Fratkin, V. Kumar, E. Foufoula-Georgiou, High spatiotemporal resolution of river planform dynamics from Landsat: The rivMAP toolbox and results from the Ucayali River. *Earth Space Sci.* **4**, 46–75 (2017).

19. E. M. Latrubesse, J. C. Stevaux, R. Sinha, Tropical rivers. *Geomorphology* **70**, 187–206 (2005).

20. E. Gautier, D. Brunstein, P. Vauchel, J. M. Jouanneau, M. Roulet, C. Garcia, J. L. Guyot, M. Castro, Channel and floodplain sediment dynamics in a reach of the tropical meandering Rio Beni (Bolivian Amazonia). *Earth Surf. Process. Landf.* **35**, 1838–1853 (2010).

21. C. Abizaid, *Floodplain Dynamics and Traditional Livelihoods in the Upper Amazon : A Study along the Central Ucayali River, Peru* (Library and Archives Canada = Bibliothèque et Archives Canada, 2008).

22. M. E. McClain, R. J. Naiman, Andean influences on the biogeochemistry and ecology of the Amazon River. *Bioscience* **58**, 325–338 (2008).

23. N. Filizola, J. L. Guyot, Suspended sediment yields in the Amazon basin: An assessment using the Brazilian national data set. *Hydrol. Process.* **23**, 3207–3215 (2009).

24. A. Devol, J. Richey, B. Forsberg, "Phosphorus in the Amazon River mainstem: Concentrations, forms and transport to the ocean," in *Teisen H, Lopez-Hernandez O, Salcedo IH, Editors. Phosphorus Cycles in Terrestrial and Aquatic Ecosystems: Regional Workshop III, Central and South America. SCOPE: Saskatchewan Institute of Pedology* (1991), pp. 9–23.

25. P. Vauchel, W. Santini, J. L. Guyot, J. S. Moquet, J. M. Martinez, J. C. Espinoza, P. Baby, O. Fuertes, L. Noriega, O. Puita, F. Sondag, P. Fraizy, E. Armijos, G. Cochonneau, F. Timouk, E. de Oliveira, N. Filizola, J. Molina, J. Ronchail, A reassessment of the suspended sediment load in the Madeira River basin from the Andes of Peru and Bolivia to the Amazon River in Brazil, based on 10 years of data from the HYBAM monitoring programme. *J. Hydrol.* **553**, 35–48 (2017).

26. M. Puhakka, R. Kalliola, M. Rajasilta, J. Salo, River types, site evolution and successional vegetation patterns in Peruvian Amazonia. *J. Biogeogr.* **19**, 651–665 (1992).

27. E. M. Latrubesse, E. Y. Arima, T. Dunne, E. Park, V. R. Baker, F. M. D'Horta, C. Wight, F. Wittmann, J. Zuanon, P. A. Baker, C. C. Ribas, R. B. Norgaard, N. Filizola, A. Ansar, B. Flyvbjerg, J. C. Stevaux, Damming the rivers of the Amazon basin. *Nature* **546**, 363–369 (2017).

28. I. A. Rivera, E. A. Cardenas, R. Espinoza-Villar, J. C. Espinoza, J. Molina-Carpio, J. M. Ayala, O. Gutierrez-Cori, J.-M. Martinez, N. Filizola, Decline of fine suspended sediments in the Madeira River basin (2003–2017). *Water* **11**, 514 (2019).

29. R. Santos, R. Pinto-Coelho, R. Fonseca, N. Simões, F. Zanchi, The decline of fisheries on the Madeira River, Brazil: The high cost of the hydroelectric dams in the Amazon Basin. *Fish. Manag. Ecol.* **25**, 380–391 (2018).

30. A. P. Lonardoni, C. P. Röpke, T. Melo, G. Torrente-Vilara, Damming in the Madeira River modifies the food spectrum of piscivorous and affects their resource partitioning. *Neotrop. Ichthyol.* **19**, e210087 (2021).

31. G. E. Petts, A. M. Gurnell, Dams and geomorphology: Research progress and future directions. *Geomorphology* **71**, 27–47 (2005).

32. B. Cluer, C. Thorne, A stream evolution model integrating habitat and ecosystem benefits. *River Res. Appl.* **30**, 135–154 (2014).

33. J. A. Nittrouer, E. Viparelli, Sand as a stable and sustainable resource for nourishing the Mississippi River delta. *Nat. Geosci.* **7**, 350–354 (2014).

34. J. Sanyal, J. Wesley Lauer, S. Kanae, Examining the downstream geomorphic impact of a large dam under climate change. *Catena* **196**, 104850 (2021).

35. A. M. Jalowska, Y. Yuan, Evaluation of SWAT impoundment modeling methods in water and sediment simulations. *J. Am. Water Resour. Assoc.* **55**, 209–227 (2019).

36. V. B. Smith, D. Mohrig, Geomorphic signature of a dammed Sandy River: The lower Trinity River downstream of Livingston Dam in Texas, USA. *Geomorphology* **297**, 122–136 (2017).

37. N. Van Manh, N. V. Dung, N. N. Hung, M. Kummu, B. Merz, H. Apel, Future sediment dynamics in the Mekong Delta floodplains: Impacts of hydropower development, climate change and sea level rise. *Glob. Planet. Change* **127**, 22–33 (2015).

38. S. Hatchard, R. J. P. Schmitt, F. Pianosi, J. Savage, P. Bates, Strategic siting and design of dams minimizes impacts on seasonal floodplain inundation. *Environ. Res. Lett.* **18**, 084011 (2023).

39. M. G. Kleinhans, W. J. McMahon, N. S. Davies, "What even is a meandering river? A philosophy-enhanced synthesis of multi-level causes and systemic interactions contributing to river meandering," in *Meandering Streamflows: Patterns and Processes across Landscapes and Scales*, A. Finotello, P. R. Durkin, Z. Sylvester, Eds. (Geological Society of London, 2024).

40.. Güneralp, J. D. Abad, G. Zolezzi, J. Hooke, Advances and challenges in meandering channels research. *Geomorphology* **163-164**, 1–9 (2012).

41. A. P. Nicholas, Modelling the continuum of river channel patterns. *Earth Surf. Process. Landf.* **38**, 1187–1196 (2013).

42. A. P. Nicholas, Modelling river and floodplain evolution using physics-based and reduced-complexity approaches. *Earth Surf. Process. Landf.* **50**, e70008 (2025).

43. G. Coco, Z. Zhou, B. van Maanen, M. Olabarrieta, R. Tinoco, I. Townend, Morphodynamics of tidal networks: Advances and challenges. *Mar. Geol.* **346**, 1–16 (2013).

44. A. B. Murray, Reducing model complexity for explanation and prediction. *Geomorphology* **90**, 178–191 (2007).

45. E. Desjardins, M. Van De Wiel, Y. Rousseau, Predicting, explaining and exploring with computer simulations in fluvial geomorphology. *Earth. Sci. Rev.* **209**, 102654 (2020).

46. J. A. Constantine, T. Dunne, J. Ahmed, C. Legleiter, E. D. Lazarus, Sediment supply as a driver of river meandering and floodplain evolution in the Amazon Basin. *Nat. Geosci.* **7**, 899–903 (2014).

47. A. C. Schwendel, A. P. Nicholas, R. E. Aalto, G. H. S. Smith, S. Buckley, Interaction between meander dynamics and floodplain heterogeneity in a large tropical sand-bed river: The Rio Beni, Bolivian Amazon. *Earth Surf. Process. Landf.* **40**, 2026–2040 (2015).

48. E. M. Latrubesse, Patterns of anabranching channels: The ultimate end-member adjustment of mega rivers. *Geomorphology* **101**, 130–145 (2008).

49. E. Wohl, B. P. Bledsoe, R. B. Jacobson, N. L. Poff, S. L. Rathburn, D. M. Walters, A. C. Wilcox, The natural sediment regime in rivers: Broadening the foundation for ecosystem management. *Bioscience* **65**, 358–371 (2015).

50. G. P. Williams, M. G. Wolman, *Downstream Effects of Dams on Alluvial Rivers* (US Government Printing Office, 1984), vol. 1286.

51. B. L. Edwards, R. F. Keim, E. L. Johnson, C. R. Hupp, S. Marre, S. L. King, Geomorphic adjustment to hydrologic modifications along a meandering river: Implications for surface flooding on a floodplain. *Geomorphology* **269**, 149–159 (2016).

52. L. A. James, Channel incision on the lower American River, California, from streamflow gage records. *Water Resour. Res.* **33**, 485–490 (1997).

53. M. Rinaldi, Recent channel adjustments in alluvial rivers of Tuscany, Central Italy. *Earth Surf. Process. Landf.* **28**, 587–608 (2003).

54. J. Li, J. Xia, Q. Ji, Rapid and long-distance channel incision in the Lower Yellow River owing to upstream damming. *Catena* **196**, 104943 (2021).

55. J. Ahmed, J. A. Constantine, T. Dunne, The role of sediment supply in the adjustment of channel sinuosity across the Amazon Basin. *Geology* **47**, 807–810 (2019).

56. N. LeRoy Poff, J. David Allan, M. B. Bain, J. R. Karr, K. L. Prestegaard, B. D. Richter, R. E. Sparks, J. C. Stromberg, The natural flow regime. *Bioscience* **47**, 769–784 (1997).

57. H. Middelkoop, A. M. Alabyan, D. B. Babich, V. V. Ivanov, "Post-dam channel and floodplain adjustment along the lower volga river, Russia," in *Geomorphic Approaches to Integrated Floodplain Management of Lowland Fluvial Systems in North America and Europe* (2015), Springer, pp. 245–264.

58. M. Tangi, S. Bizzi, R. Schmitt, A. Castelletti, Balancing sediment connectivity and energy production via optimized reservoir sediment management strategies. *Water Resour. Res.* **59**, e2022WR034033 (2023).

59. P. G. Buma, J. C. Day, Channel morphology below reservoir storage projects. *Environ. Conserv.* **4**, 279–284 (1977).

60. G. Grill, B. Lehner, M. Thieme, B. Geenen, D. Tickner, F. Antonelli, S. Babu, P. Borrelli, L. Cheng, H. Crochetiere, H. Ehalt Macedo, R. Filgueiras, M. Goichot, J. Higgins, Z. Hogan, B. Lip, M. E. McClain, J. Meng, M. Mulligan, C. Nilsson, J. D. Olden, J. J. Opperman, P. Petry, C. Reidy Liermann, L. Sáenz, S. Salinas-Rodríguez, P. Schelle, R. J. P. Schmitt, J. Snider, F. Tan, K. Tockner, P. H. Valdujo, A. van Soesbergen, C. Zarfl, Mapping the world's free-flowing rivers. *Nature* **569**, 215–221 (2019).

61. R. H. Meade, J. A. Moody, Causes for the decline of suspended-sediment discharge in the Mississippi River system, 1940-2007. *Hydrol. Process.* **24**, 35–49 (2010).

62. D. A. Edmonds, S. C. Toby, C. G. Siverd, R. Twilley, S. J. Bentley, S. Hagen, K. Xu, Land loss due to human-altered sediment budget in the Mississippi River Delta. *Nat. Sustain.* **6**, 644–651 (2023).

63. F. M. Holly, M. F. Karim, Simulation of Missouri River bed degradation. *J. Hydraul. Eng.* **112**, 497–516 (1986).

64. A. T. Zhang, V. X. Gu, Global Dam Tracker: A database of more than 35,000 dams with location, catchment, and attribute information. *Sci. Data* **10**, 111 (2023).

65. F. J. Magilligan, K. H. Nislow, Long-term changes in regional hydrologic regime following impoundment in a humid-climate watershed. *J. Am. Water Resour. Assoc.* **37**, 1551–1569 (2001).

66. W. Junk, P. Bayley, R. Sparks, The flood pulse concept in river-floodplain systems. *Can. Spec. Publ. Fish. Aquat. Sci.* **106**, 110–127 (1989).

67. J. N. Cotta, Contributions of local floodplain resources to livelihoods and household income in the Peruvian Amazon. *Forest Policy Econ.* **59**, 35–46 (2015).

68. H. ter Steege, N. C. A. Pitman, D. Sabatier, C. Baraloto, R. P. Salomão, J. E. Guevara, O. L. Phillips, C. V. Castilho, W. E. Magnusson, J.-F. Molino, A. Monteagudo, P. N. Vargas, J. C. Montero, T. R. Feldpausch, E. N. H. Coronado, T. J. Killeen, B. Mostacedo, R. Vasquez, R. L. Assis, J. Terborgh, F. Wittmann, A. Andrade, W. F. Laurance, S. G. W. Laurance, B. S. Marimon, B.-H. Marimon, I. C. G. Vieira, I. L. Amaral, R. Brienen, H. Castellanos, D. C. López, J. F. Duivenvoorden, H. F. Mogollón, F. D. de Almeida Matos, N. Dávila, R. García-Villacorta, P. R. S. Diaz, F. Costa, T. Emilio, C. Levis, J. Schietti, P. Souza, A. Alonso, F. Dallmeier, A. J. D. Montoya, M. T. F. Piedade, A. Araujo-Murakami, L. Arroyo, R. Gribel, P. V. A. Fine, C. A. Peres, M. Toledo, G. A. Aymard C, T. R. Baker, C. Cerón, J. Engel, T. W. Henkel, P. Maas, P. Petronelli, J. Stropp, C. E. Zartman, D. Daly, D. Neill, M. Silveira, M. R. Paredes, J. Chave, D. de Andrade Lima Filho, P. M. Jørgensen, A. Fuentes, J. Schöngart, F. C. Valverde, A. Di Fiore, E. M. Jimenez, M. C. P. Mora, J. F. Phillips, G. Rivas, T. R. van Andel, P. von Hildebrand, B. Hoffman, E. L. Zent, Y. Malhi, A. Prieto, A. Rudas, A. R. Ruschell, N. Silva, V. Vos, S. Zent, A. A. Oliveira, A. C. Schutz, T. Gonzales, M. T. Nascimento, H.

Ramirez-Angulo, R. Sierra, M. Tirado, M. N. U. Medina, G. van der Heijden, C. I. A. Vela, E. V. Torre, C. Vriesendorp, O. Wang, K. R. Young, C. Baider, H. Balslev, C. Ferreira, I. Mesones, A. Torres-Lezama, L. E. U. Giraldo, R. Zagt, M. N. Alexiades, L. Hernandez, I. Huamantupa-Chuquimaco, W. Milliken, W. P. Cuenca, D. Pauletto, E. V. Sandoval, L. V. Gamarra, K. G. Dexter, K. Feeley, G. Lopez-Gonzalez, M. R. Silman, Hyperdominance in the Amazonian Tree Flora. *Science* **342**, 1243092 (2013).

69. S. B. Correa, K. V. Coronado-Franco, C. Jézéquel, A. Cantarute Rodrigues, K. O. Evans, J. J. Granger, H. Ter Steege, I. Leão do Amaral, L. de Souza Coelho, F. Wittmann, F. D. de Almeida Matos, D. de Andrade Lima Filho, R. P. Salomão, C. V. Castilho, J. E. Guevara, M. de J. Veiga Carim, O. L. Phillips, M. T. Fernandez Piedade, L. O. Demarchi, J. Schöngart, J. D. Cardenas Revilla, M. Pires Martins, M. V. Irume, J. R. da Silva Guimarães, J. Ferreira Ramos, A. Costa Quaresma, N. C. A. Pitman, B. G. Luize, E. M. Moraes de Leão Novo, E. Martins Venticinque, T. Sanna Freire Silva, P. Núñez Vargas, A. G. Manzatto, N. Farias Costa Reis, J. Terborgh, K. R. Casula, E. N. Honorio Coronado, J. C. Montero, A. Monteagudo Mendoza, T. R. Feldpausch, F. Machado Durgante, N. Castaño Arboleda, B. S. Marimon, B. H. Marimon-Junior, T. J. Killeen, R. Vasquez, B. Mostacedo, R. L. Assis, D. Dantas do Amaral, J. E. Householder, M. F. Simon, M. Brilhante de Medeiros, H. Lima de Queiroz, M. A. Lopes, J. L. Lima Magalhães, P. R. Stevenson, B. Barçante Ladvocat Cintra, A. Araujo-Murakami, T. R. Baker, Y. Oliveira Feitosa, H. F. Mogollón, J. F. Duivenvoorden, L. V. Ferreira, J. J. de Toledo, J. A. Comiskey, A. Lopes, G. Damasco, A. Vicentini, F. Cornejo Valverde, V. H. F. Gomes, A. Alonso, F. Dallmeier, D. P. P. de Aguiar, R. Gribel, J. C. Licona, B. E. Villa Zegarra, M. Carneiro Guedes, C. Cerón, R. Thomas, W. Milliken, W. Campelo, B. W. Albuquerque, B. Klitgaard, J. S. Tello, A. Fuentes Claros, G. Rivas-Torres, J. F. Phillips, P. von Hildebrand, T. Gonzales, C. I. A. Vela, B. Hoffman, B. M. Flores, M. M. Pombo, M. Rocha, M. Holmgren, A. Cano, M. N. Umaña, L. F. Casas, H. Balslev, L. E. Urrego Giraldo, R. Bigorne, T. Oberdorff, J. A. Maldonado-Ocampo, H. Ortega, M. Hidalgo, K. Martens, G. Torrente-Vilara, J. Zuanon, A. Acosta, E. Agudelo, S. Barrera Maure, D. A. Bastos, J. Bogotá Gregory, F. G. Cabeceira, A. L. C. Canto, F. M. Carvajal-Vallejos, L. N. Carvalho, A. Cella-Ribeiro, R. Covain, M. S. Dias, C. Donascimiento, C. R. C. Dória, C. Duarte, E. J. G. Ferreira, A. V. Galuch, T. Giarrizzo, R. P. Leitão, J. G. Lundberg, M. Maldonado, J. I. Mojica, L. F. A. Montag, W. Ohara, T. H. S. Pires, M. Pouilly, S. Prada-Pedreros, L. J. de Queiroz, L. Rapp Py-Daniel, F. R. V. Ribeiro, R. Ríos Herrera, M. Rodrigues Dos Anjos, I. H. Lourenco,

J. Sarmiento, L. M. Sousa, L. F. Stegmann, J. Valdiviezo-Rivera, F. Villa, T. Yunoki, P. A. Tedesco, Floodplain forests drive fruit-eating fish diversity at the Amazon Basin-scale. *Proc. Natl. Acad. Sci. U.S.A.* **122**, e2414416122 (2025).

70. M. A. Palmer, E. S. Bernhardt, J. D. Allan, P. S. Lake, G. Alexander, S. Brooks, J. Carr, S. Clayton, C. N. Dahm, J. Follstad Shah, D. L. Galat, S. G. Loss, P. Goodwin, D. D. Hart, B. Hassett, R. Jenkinson, G. M. Kondolf, R. Lave, J. L. Meyer, T. K. O'Donnell, L. Pagano, E. Sudduth, Standards for ecologically successful river restoration. *J. Appl. Ecol.* **42**, 208–217 (2005).

71. S. Chaudhari, E. Brown, R. Quispe-Abad, E. Moran, N. Müller, Y. Pokhrel, In-stream turbines for rethinking hydropower development in the Amazon basin. *Nat. Sustain.* **4**, 680–687 (2021).

72. R. Ranasinghe, C. Swinkels, A. Luijendijk, D. Roelvink, J. Bosboom, M. Stive, D. J. Walstra, Morphodynamic upscaling with the MORFAC approach: Dependencies and sensitivities. *Coast. Eng.* **58**, 806–811 (2011).

73. R. J. Boothroyd, R. D. Williams, T. B. Hoey, B. Barrett, O. A. Prasojo, Applications of Google Earth Engine in fluvial geomorphology for detecting river channel change. *Wiley Interdiscip. Rev. Water* **8**, e21496 (2021).

74. M. Z. M. Brückner, A. P. Nicholas, R. Aalto, R. Paes de Almeida, P. Ashworth, J. Best, T. Dunne, M. Ianniruberto, *Hydro-morphodynamic Data from Numerical Simulations of Different Dam Scenarios in Andean Rivers of the Amazon Basin* (NERC EDS Environmental Information Data Centre, 2026).

75. P. D. Bates, M. S. Horritt, T. J. Fewtrell, A simple inertial formulation of the shallow water equations for efficient two-dimensional flood inundation modelling. *J. Hydrol.* **387**, 33–45 (2010).

76. S. Ikeda, Lateral bed load transport on side slopes. *J. Hydraul. Div.* **108**, 1369–1373 (1982).

77. L. C. Van Rijn, *Principles of Sediment Transport in Rivers, Estuaries and Coastal Seas* (Aqua Publications, 1993), vol. 1006.

78. A. W. Baar, M. Boechat Albernaz, W. M. van Dijk, M. G. Kleinhans, Critical dependence of morphodynamic models of fluvial and tidal systems on empirical downslope sediment transport. *Nat. Commun.* **10**, 4903 (2019).

79. P. Parolin, F. Wittmann, Struggle in the flood: Tree responses to flooding stress in four tropical floodplain systems. *AoB Plants* **2010**, plq003 (2010).

80. L. A. K. Mertes, T. Dunne, L. A. Martinelli, Channel-floodplain geomorphology along the Solimões-Amazon River, Brazil. *GSA Bull.* **108**, 1089–1107 (1996).

81. C. Jézéquel, T. Oberdorff, P. A. Tedesco, L. Schmitt, Geomorphological diversity of rivers in the Amazon Basin. *Geomorphology* **400**, 108078 (2022).

82. J. A. Morgan, N. Kumar, A. R. Horner-Devine, S. Ahrendt, E. Istanbullouglu, C. Bandaragoda, The use of a morphological acceleration factor in the simulation of large-scale fluvial morphodynamics. *Geomorphology* **356**, 107088 (2020).

83. W. V. Manyari, O. A. de Carvalho Jr., Environmental considerations in energy planning for the Amazon region: Downstream effects of dams. *Energy Policy* **35**, 6526–6534 (2007).
